# Virulence Regulation with Venus Flytrap Domains: Structure and Function of the Periplasmic Moiety of the Sensor-Kinase BvgS

**DOI:** 10.1371/journal.ppat.1004700

**Published:** 2015-03-04

**Authors:** Elian Dupré, Julien Herrou, Marc F. Lensink, René Wintjens, Alexey Vagin, Andrey Lebedev, Sean Crosson, Vincent Villeret, Camille Locht, Rudy Antoine, Françoise Jacob-Dubuisson

**Affiliations:** 1 Center for Infection and Immunity (CIIL), Institut Pasteur de Lille, Lille, France; 2 Center for Infection and Immunity (CIIL), University Lille North of France, Lille, France; 3 UMR 8204, Centre National de la Recherche Scientifique (CNRS), Lille, France; 4 U1019, Institut National de la Santé et de la Recherche Médicale (INSERM), Lille, France; 5 Unité de Glycobiologie Structurale et Fonctionnelle, CNRS UMR8576, University Lille North of France, Villeneuve d’Ascq, France; 6 Laboratory of Biopolymers and Supramolecular Nanomaterials, Université Libre de Bruxelles, Brussels, Belgium; 7 Structural Biology Laboratory, University of York, York, England, United Kingdom; 8 Research Complex at Harwell, Science and Technology Facilities Council Rutherford Appleton Laboratory, Didcot, England, United Kingdom; 9 Department of Biochemistry & Molecular Biology, University of Chicago, Chicago, Illinois, United States of America; Yale University School of Medicine, UNITED STATES

## Abstract

Two-component systems (TCS) represent major signal-transduction pathways for adaptation to environmental conditions, and regulate many aspects of bacterial physiology. In the whooping cough agent *Bordetella pertussis*, the TCS BvgAS controls the virulence regulon, and is therefore critical for pathogenicity. BvgS is a prototypical TCS sensor-kinase with tandem periplasmic Venus flytrap (VFT) domains. VFT are bi-lobed domains that typically close around specific ligands using clamshell motions. We report the X-ray structure of the periplasmic moiety of BvgS, an intricate homodimer with a novel architecture. By combining site-directed mutagenesis, functional analyses and molecular modeling, we show that the conformation of the periplasmic moiety determines the state of BvgS activity. The intertwined structure of the periplasmic portion and the different conformation and dynamics of its mobile, membrane-distal VFT1 domains, and closed, membrane-proximal VFT2 domains, exert a conformational strain onto the transmembrane helices, which sets the cytoplasmic moiety in a kinase-on state by default corresponding to the virulent phase of the bacterium. Signaling the presence of negative signals perceived by the periplasmic domains implies a shift of BvgS to a distinct state of conformation and activity, corresponding to the avirulent phase. The response to negative modulation depends on the integrity of the periplasmic dimer, indicating that the shift to the kinase-off state implies a concerted conformational transition. This work lays the bases to understand virulence regulation in *Bordetella*. As homologous sensor-kinases control virulence features of diverse bacterial pathogens, the BvgS structure and mechanism may pave the way for new modes of targeted therapeutic interventions.

## Author Summary

Bacteria make use of two-component transduction systems, composed of a sensor-kinase and a response regulator, to perceive environmental signals and orchestrate an appropriate response. The virulence regulon of the whooping cough agent *Bordetella pertussis* is controlled by the two-component system BvgAS. The sensor-kinase BvgS harbor extra-cytoplasmic Venus flytrap perception domains similar to those found in neuronal receptors, and it is the prototype of a large bacterial protein family. We report the atomic structure of the extra-cytoplasmic moiety of BvgS, which shows a novel dimeric arrangement. We show that the virulent phase of *B*. *pertussis* that occurs by default corresponds to a specific conformation of BvgS generated by the periplasmic architecture itself and by the differential dynamics of its Venus flytrap domains. The perception of negative signals by the periplasmic domains causes BvgS to shift to a different conformation that corresponds to the avirulent phase of the bacteria. In addition to contributing to our understanding of virulence regulation by *B*. *pertussis* at a time of whooping cough re-emergence, this study also paves the way to the mechanistic exploration of the homologous sensor-kinases found in various bacterial pathogens.

## Introduction

Two-component sensory transduction systems (TCSs) regulate various physiological processes in response to environmental changes [1]. They are abundant throughout the phylogenetic tree except for vertebrates and represent major bacterial signaling pathways [2,3]. TCSs notably regulate the cell cycle, motility, biofilm formation or antibiotic resistance, as well as the virulence of major pathogens [4–8]. TCSs are typically composed of a sensor-kinase activated by environmental stimuli and a response regulator mediating phosphorylation-dependent effects [9,10]. Upon perception of a physical or chemical signal, auto-phosphorylation of a conserved cytoplasmic His residue of the sensor-kinase is followed by transfer of the phosphoryl group to a conserved Asp residue of the response regulator. The phosphorylated response regulator mediates a specific, frequently transcriptional, cellular response [11]. There is considerable diversity among TCSs regarding domain composition and organization [9,10].

**Fig 1:**
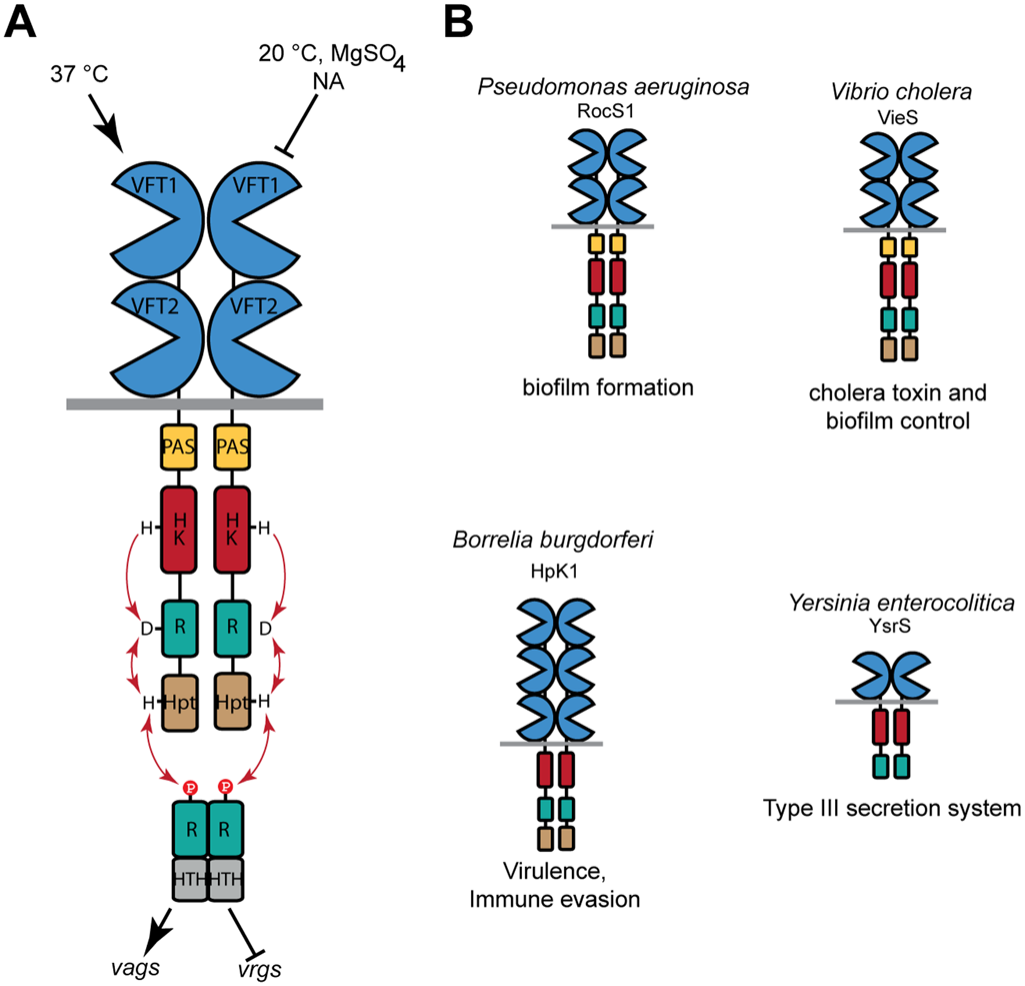
Function of BvgS and selected homologs. A. Schematic representation of virulence regulation by BvgAS in *B*. *pertussis*. Only the virulent (Bvg^+^) and avirulent (Bvg^-^) phases of the bacterium are represented for simplicity. Conditions that turn the bacteria to the avirulent phase include low temperatures and negative modulators such as sulfate or nicotinate (NA) ions. The *vags* (virulence-activated genes) are trans-activated by phosphorylated BvgA, while the *vrgs* (virulence-repressed genes) are upregulated in the avirulent phase. An intermediate phase occurs at low modulator concentrations (see text). From N to C terminus, 135 kDa-BvgS is composed of two periplasmic VFT domains, a transmembrane segment, a PAS domain, followed by a histidine-kinase (HK), a receiver (R) and a Histidine phosphotransfer (Hpt) domains that make up a phosphorelay (represented by arrows). BvgA is composed of a receiver domain and a helix-turn-helix DNA-binding domain (HTH). B. Structural organization of selected BvgS homologs, with the same color code as for BvgS. Note that the domain composition varies in the family. The cellular functions regulated by these sensor-kinases are also indicated.

*Bordetella pertussis*, the whooping cough agent, colonizes the upper respiratory tract of humans [12]. Transcription of its virulence regulon is positively regulated by the TCS BvgAS [13]. Over one hundred genes belong to the Bvg regulon, including those coding for the adhesins and toxins and their secretion and assembly machineries [14]. The virulent, Bvg^+^ phase, in which phosphorylated BvgA trans-activates the expression of the virulence regulon, is essential for the development of the infection cycle of *B*. *pertussis* and other pathogenic *Bordetella* species [13,15]. The kinase and phosphotransfer activities of BvgS are maximal (referred to below as the ‘kinase-on’ state) without specific chemical stimuli and at 37°C, the *B*. *pertussis* host body temperature, while low temperatures and specific negative modulators turn these activities off in laboratory conditions (referred to below as the ‘kinase-off’ state). Thus, millimolar concentrations of nicotinate or sulfate ions result in the dephosphorylation of BvgA, switching the bacteria to the avirulent, Bvg^-^ phase [16,17]. Virulence genes are no longer expressed, while a smaller set of virulence-repressed genes (*vrg*s) are upregulated [18,19]. At low modulator concentrations, an intermediate Bvg^i^ phase occurs in which the reduced concentration of phosphorylated BvgA is sufficient to transactivate ‘early’ virulence genes as well as specific intermediate genes [13,20,21]. Thus, BvgAS operates like a rheostat, determining several states of gene expression that might correspond to distinct temporal or spatial situations in the course of infection. BvgS is composed of periplasmic Venus flytrap (VFT) domains, a transmembrane segment, a PAS domain, and a kinase and additional domains that make up a phosphorelay (Fig. 1A). The cytoplasmic moiety of BvgS dimerizes, similar to the other TCS sensor-kinases [22,23].

BvgS is the prototype of a family of bacterial VFT-domain-containing sensor-kinases [24]. VFT domains have a bi-lobed structure with two mobile jaws delimitating a putative ligand-binding cavity [25,26]. They exist in open and closed conformations that interconvert by clamshell motions. Typically, binding of a ligand in the cavity stabilizes the closed conformation, which triggers downstream cellular events such as transport or signaling. The periplasmic moiety of BvgS is composed of two VFT domains, membrane-distal VFT1 and membrane-proximal VFT2. We have previously reported the structure of the isolated VFT2 domain and showed that nicotinate and related negative modulators bind to VFT2 [27]. There are currently more than 2000 predicted BvgS homologs, containing from one to five VFT domains. Some of them are found in major pathogens, including *Pseudomonas aeruginosa*, *Vibrio cholerae*, *Yersinia enterocolitica* and *Borrelia burgdorferi*, in which they regulate various responses that contribute to pathogenicity [28–32] (Fig. 1B). Unlike those of classical TCSs, the molecular mechanisms of signal perception and transduction by these VFT-containing sensor-kinases are largely unknown.

In this work, we describe the structure of the periplasmic portion of BvgS, revealing a novel homo-dimeric architecture with two highly intricate polypeptide chains wound around each other. A combination of site-directed mutagenesis, functional analyses *in vivo* and molecular modeling indicated that the integrity of the periplasmic domain is necessary both to maintain BvgS in a kinase-on state by default and to bring about conformational changes that switch the protein to the kinase-off state in response to negative modulation. This study shows that BvgS represents a new paradigm of bacterial two-component sensor-kinases and contributes to our understanding of virulence regulation in *Bordetella*.

## Results

### Structure of the periplasmic domain of BvgS

**Fig 2:**
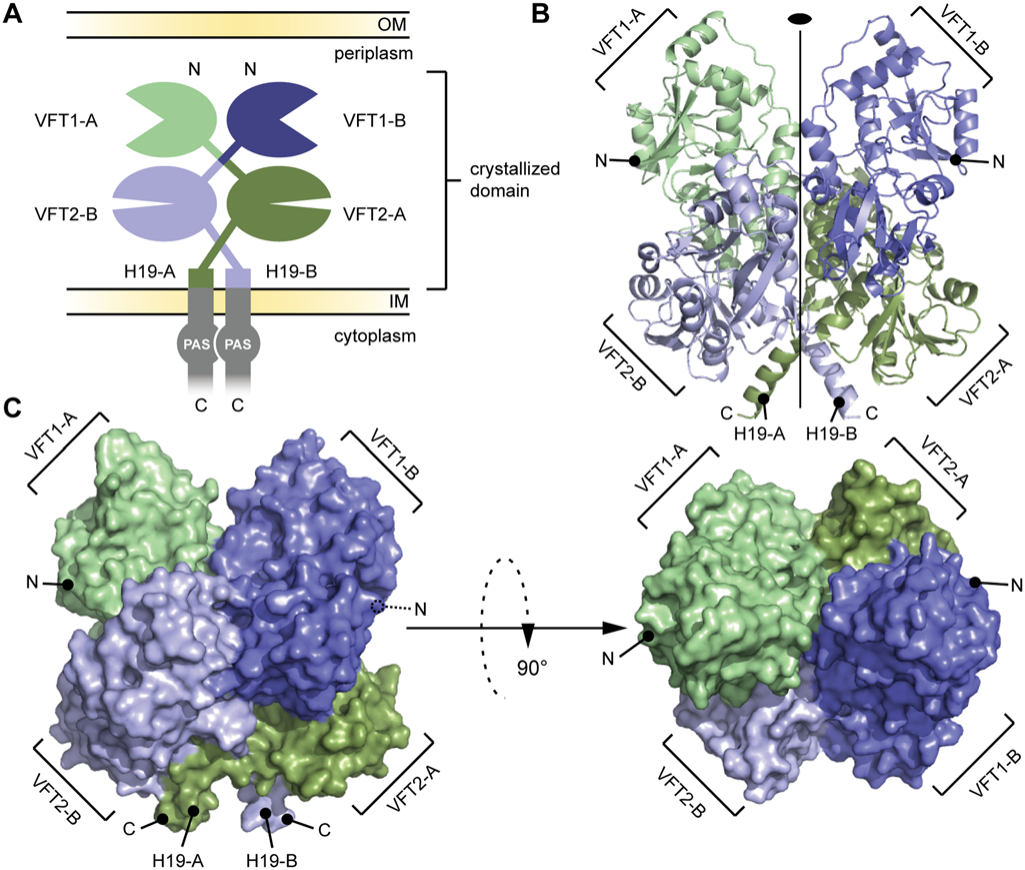
General organization of the BvgS periplasmic domain. A. Schematic representation of the homodimeric BvgS periplasmic portion. The protomers A and B are shown in shades of green and blue, respectively. One protomer consists of two VFT domains and a C-terminal H19 α helix. B. Ribbon representation of the X-ray structure of the BvgS periplasmic domain, the same color code as in (A) is used to show the different VFTs. The two-fold symmetry axis is indicated. C. Surface representation of the periplasmic domain of BvgS. On the left, the view angle is similar to (B), while on the right, a 90° clockwise rotation along the x-axis was applied. N and C denote the N and C termini of the two protomers.

The periplasmic domain of BvgS (residues Ala_29_-Leu_544_, which includes VFT1 and VFT2) was produced in *Escherichia coli* and crystallized as a recombinant protein with a 60-residue-long GB1 domain at the N terminus and a 6-His tag at the C terminus. The structure was solved to a resolution of 3.1 Å (Fig. 2, S1 Table). BvgS forms intricate butterfly-shaped dimers in which the A and B polypeptide chains (‘protomers’) wind around each other, with an extensive dimeric interface of ≈ 4000 Å^2^. The two protomers overlap with an RMSD of 1.184 Å. The N-terminal GB1 domain and C-terminal His tag are not visible in the electron density maps.

A two-fold symmetry axis runs parallel to the long axis of the BvgS dimer. The N termini of the two protomers are located on the outer surface of the dimer, and their C termini interrupt α helices at the membrane-proximal end of the structure. VFT1 and VFT2 adopt typical Venus flytrap architectures consisting of two α/β subdomains called lobes 1 and 2 (hereafter L1 and L2) separated by a cleft. They have similar topologies with two crossings between the lobes (S1 Fig). The hinge is formed of anti-parallel β strands in VFT2 and flexible loops in VFT1. The VFT2s are followed by the C-terminal (Ct) domains that encompass the Gly_527_-Pro_532_ Ct loops and the H19 Ct helices (Figs. 2 and S1). In the absence of membrane constraints, the H19s adopt divergent orientations in the crystal structure. In full-length BvgS they are predicted to continue across the membrane down to the cytoplasmic PAS domain, with a total length of 60 residues.

**Fig 3:**
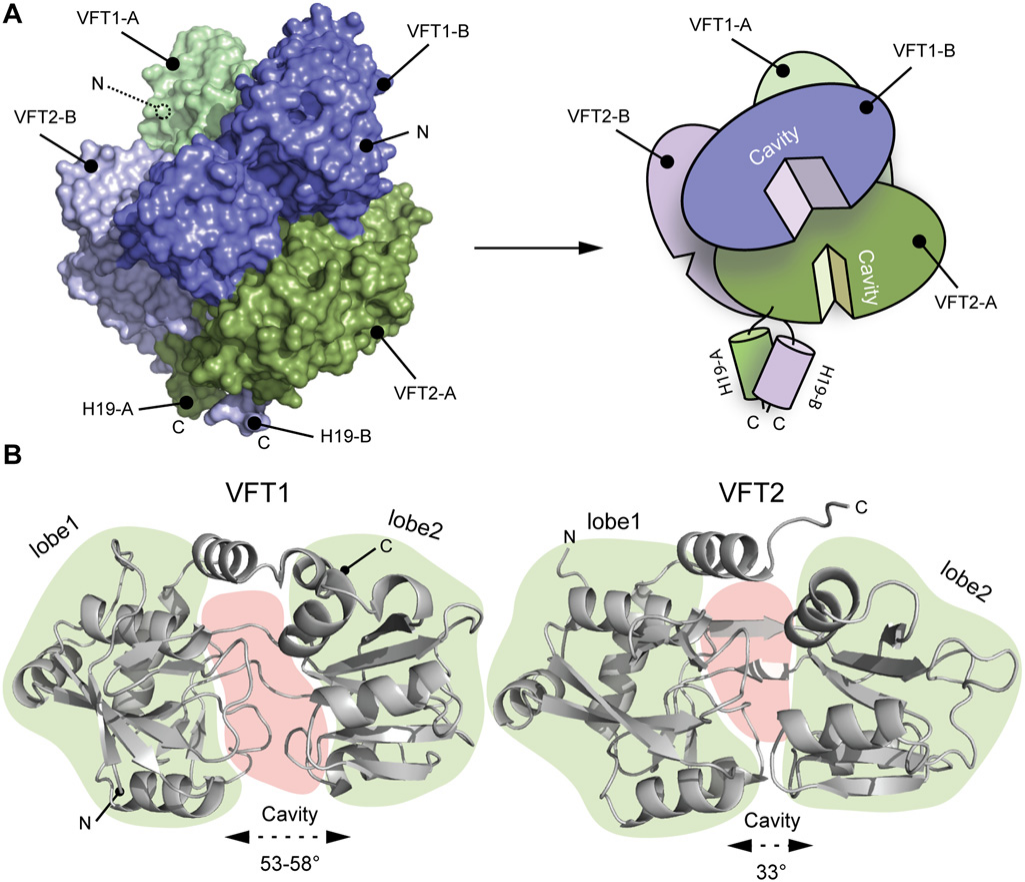
Characterization of the VFT domains. A. Surface and cartoon representation of BvgS showing that VFT1-B is open and VFT2-A is in an apo-closed conformation. B. Ribbon representation of the open VFT1 and closed VFT2 domains. The lobes are delimited in light green and the cavities in light red. The opening angles for the VFTs are given. The linker (H9) joining VFT1 and VFT2 and the Ct loop that follows VFT2 have been included in the representation of the VFT1 and VFT2 domains, respectively. N and C indicate the N and C termini of each protomer (in A) or VFT domain (in B).

The two VFT1s are open, while atypically the VFT2s are closed with no ligand in their inter-lobe cavities (Fig. 3), consistent with the structure of VFT2 alone [27]. The VFT1 cavities are each oriented toward the hinge of the VFT2 domain of the other protomer, and the cavities of the VFT2s are each oriented toward the H19 helix of the opposite protomer (Fig. 3).

**Fig 4:**
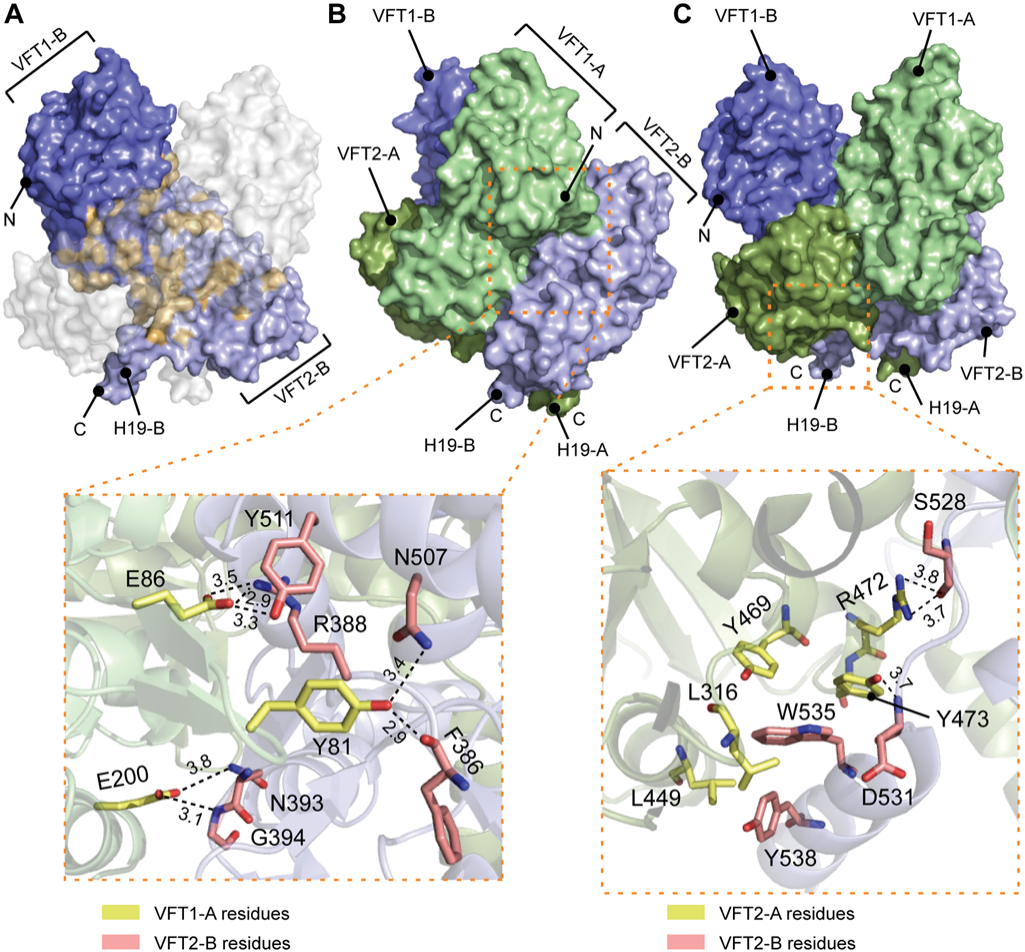
Interfaces between the VFT domains important for the kinase-on state. A. Surface representation of protomer B (in blue); the residues interacting with protomer A are shown in orange. To help visualizing these interactions, a “ghost” protomer A is represented in transparent white on top of protomer B. B. Illustration of the VFT1-VFT2 inter-protomer interface. A side view of BvgS is shown in surface representation, with the VFT1 of one protomer in green and the VFT2 of the other protomer in pale blue. A zoom delimited by a dashed orange box shows specific residues that are critical for BvgS function, as shown by mutagenesis. The side chains of Tyr_81_ and Glu_86_ of the β hairpin in VFT1_L1_ form hydrogen bonds with Phe_386_ and Arg_388_ at one extremity of the VFT2 hinge, and with residues of the α helix H17. Glu_200_ belongs to VFT1_L2_, and its side chain makes hydrogen bonds with Asn_393_ and Gly_394_ at the other extremity of the VFT2 hinge. C. Illustration of the VFT2-Ct domain inter-protomer interface. In the upper panel, BvgS is shown in surface representation, with protomer A in green and protomer B in blue. A zoom shows specific residues involved in critical interactions for BvgS kinase activity. Thus, Trp_535_ from H19 stacks in a hydrophobic and aromatic pocket mainly lined with VFT2_L2_ residues of the other protomer, and Arg_472_ and Tyr_473_ from helix H16 in VFT2_L2_ interact with Ser_528_ and Asp_531_ in the Ct loop of the other protomer. Hydrogen-bond distances are reported in angstroms.

The VFT1_L1s_ interact with each other through several hydrogen bonds between their H8s, while the VFT2s are not directly interconnected. Both lobes of the VFT1s, VFT1_L1_ and VFT1_L2_, contact the hinge and lobes of VFT2 of the opposite protomer (Fig. 4), forming the largest dimeric interfaces. Other large interfaces occur between VFT1_L2_ and VFT2 of the same protomer, and between VFT2_L2_ and the Ct domains. In particular, both the Ct loop and the N terminus of H19 strongly interact with VFT2_L2_ of the opposite protomer through hydrogen bonds and through π-stacking interactions that involve a conserved residue in the BvgS family, Trp_535_ (Fig. 4).

### Conformation and dynamics of the VFT domains

In the crystal structure, the VFT1 domains are open and unliganded, while conversely the VFT2 domains are closed without ligands. We performed normal mode analyses of BvgS motions based on a Gaussian network model to identify the main global motions that are accessible to the protein based on its tridimensional structure. The first, lowest-frequency normal modes are usually most relevant to function. For BvgS, the first two modes of motion consist of large motions of one VFT1_L1_ (S2 Fig). In contrast, the VFT2s move together as a rigid body, as shown in mode #3. Mode #4 consists of motions of both VFT1_L2s_ together with the VFT2s. Thus, the first lobes of the VFT1s in particular can make large motions, while the VFT2 motions are more restrained and mainly coupled to each other and to those of the VFT1s. This was confirmed by performing molecular dynamics simulations to measure the evolution of the opening angles of the VFTs over time. In the first parts of the simulations, the VFT1s make clamshell motions, while motions of the VFT2s are limited around their closed conformations (S2 Fig). As the simulations progress the VFT1 mobility is reduced, which suggests that sustained VFT motions may require the feedback from the transmembrane and cytoplasmic portions of BvgS absent from our model. These *in silico* analyses thus indicate that the X-ray structure reflects *bona fide* differences between the VFT1 and VFT2 domains in terms of conformation and dynamics.

**Fig 5:**
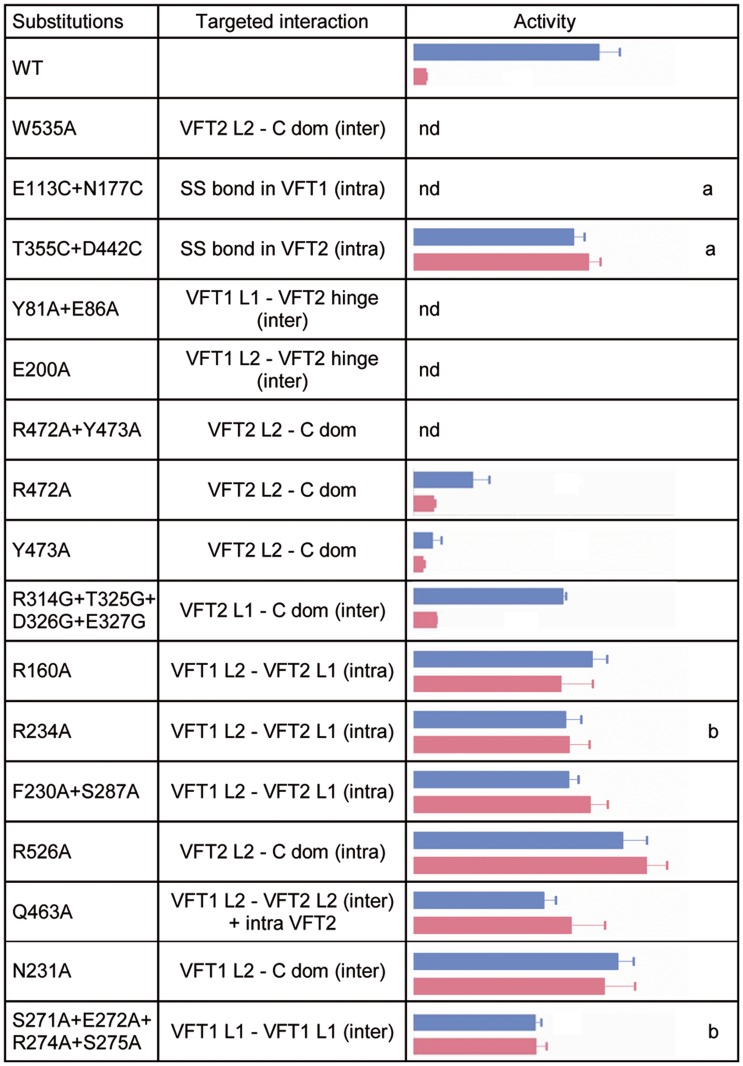
*In vivo* effects of the substitutions in BvgS. A *lacZ* reporter gene under the control of the Bvg-regulated *ptx* promoter was used for determination of BvgS kinase activity in standard or modulated culture conditions. Blue and pink bars indicate kinase activity levels of bacteria producing the indicated BvgS variants and grown without or with 8 mM nicotinate, respectively, with the standard errors of the mean calculated from three distinct experiments. The middle column indicates the interfaces in which the targeted interactions are located, with inter- and intra-protomer interfaces designated ‘inter’ and ‘intra’, respectively. Nd, no β-gal activity detected; a, wild type activity and/or modulation recovered when cells were grown in the presence of TCEP; b, BvgS variants only responsive to high nicotinate concentrations (20 mM). The full set of data is shown in S5 Fig.

We then asked whether VFT1 closing—as might happen upon binding of a ligand—would affect BvgS activity. We locked the VFT1 domains in closed conformations by generating a disulfide (S-S) bonds across their cavity [33,34]. Two residues located on the edges of the lobes were replaced by Cys to obtain BvgS_E113C+N177C_ (S3 Fig). The corresponding point mutations were inserted into the chromosomal *bvg* locus by allelic exchange, and we verified the production of the protein and the formation of the S-S bond by immunoblotting (S4 Fig). The *in vivo* effect of the substitution on BvgS function was then measured by using a reporter system with the *lacZ* gene under the control of the Bvg-regulated *ptx* promoter [35]. *In vivo* formation of the S-S bond in VFT1 abrogates the kinase activity of BvgS (Fig. 5). This phenotype is reverted by the addition of a reducing agent, TCEP, to the growth medium (S5 Fig), which confirms that the S-S bond forms *in vivo* and shows that the loss of function is related to its presence and not to the Cys substitutions.

The VFT2s remain closed even when isolated [27]. Nevertheless, to maintain them closed *in vivo* we also generated an S-S bond between their lobes using a similar method as above, yielding BvgS_T355C+D442C_. We checked that the S-S bond was formed (S4 Fig; see also below). In contrast to VFT1, closing VFT2 was found to have no effect on the BvgS kinase activity as determined with the *ptx-lacZ* reporter (Figs. 5 and S5). Altogether thus, closing of the VFT1 domains and/or restraining their mobility abrogate BvgS kinase activity. In contrast, closed VFT2 domains correspond to the kinase-on state of BvgS. The different conformations and dynamics of the two VFT domains thus contribute to BvgS function.

### Importance of periplasmic domain integrity for BvgS kinase activity

*B*. *pertussis* is in the virulent, Bvg^+^ phase by default at 37°C. To determine the role of the periplasmic domain of BvgS in maintaining this kinase-on state, we loosened the connections between the periplasmic and cytoplasmic moieties of BvgS by replacing Trp_535_ with Ala. This residue is located in the C-terminal helix H19 and it contributes to connecting each H19 to the VFT2_L2_ of the opposite protomer (Fig. 4C). After allelic exchange, the effect of the substitution on BvgS function was measured by using the *ptx-lacZ* reporter system. The BvgS_W535A_ variant has no kinase activity (Fig. 5). The presence of BvgS_W535A_ in *B*. *pertussis* membranes was verified, showing that the substitution does not affect the structure of the protein in such a way as to prevent its integration in the membrane or to cause its proteolytic degradation *in vivo* (S4 Fig). Thus, the kinase-on state of BvgS depends on tight connections between the periplasmic domains and the transmembrane H19 helices.

To confirm that the periplasmic portion imposes a specific conformation on the cytoplasmic moiety, we introduced other substitutions in the inter-protomer interfaces between the VFT2s and the Ct domains, by targeting residues whose side chains connect the VFT2_L2s_ and the Ct loops that precede the H19s (Fig. 4C). Thus, Arg_472_ and Tyr_473_ located in helix H16 of VFT2_L2_ form hydrogen bonds with residues of the Ct loop of the other protomer. Their simultaneous replacement by Ala abolishes BvgS kinase activity, while the single-substitution variants BvgS_R472A_ and BvgS_Y473A_ are partially active (Figs. 5 and S5). This indicates that the inter-protomer interface between H16 in VFT2 and the Ct loop is critical and that it is maintained by partly redundant interactions. In contrast, substitutions at the tip of a β hairpin in VFT2_L1_ whose residues interact with the other face of the Ct loop do not affect BvgS function, as shown with BvgS_R324G/T325G/D326G/E327G_. The effect of disrupting of specific interactions between the VFT2_L2s_ and the Ct loops preceding the H19s is consistent with the effect of the W_535_A substitution, showing that the kinase-on state depends on VFT2-Ct domain inter-protomer connections.

To identify additional architectural features of the periplasmic dimer critical to maintain BvgS in its kinase-on state, we disrupted specific interactions in other intra-dimer interfaces of BvgS by site-directed mutagenesis. We targeted residues in the large interfaces between the VFT1s and the VFT2s of the opposite protomers (Fig. 4B). The side chains of Tyr_81_ and Glu_86_ in a β hairpin of VFT1_L1_ and that of Glu_200_ in helix H5 of VFT1_L2_ form hydrogen bonds with residues at the N- and C-terminal sides of the first hinge strand of VFT2, respectively. Two BvgS variants, BvgS_Y81A+E86A_ and BvgS_E200A_ were generated and analyzed as above (Figs. 5, S4 and S5). Neither of them is functional, demonstrating that connections between the two lobes of VFT1 and the hinge of VFT2 of the opposite protomer are essential to maintain the kinase-on state of BvgS. In contrast, the replacement of Gln_463_ by Ala in the same large inter-protomer VFT1-VFT2 interface does not affect activity (Figs. 5 and S5). Gln_463_ is part of VFT2 but not located in the hinge, unlike the residues of VFT2 in contact with Tyr_81_, Glu_86_ and Glu_200_. The loss of kinase activity of the BvgS_Y81A+E86A_ and BvgS_E200A_ variants might result from the loss of constraints applied by the VFT1 lobes on the VFT2 hinge.

In contrast, disruption of specific interactions in other dimeric interfaces (S3 Fig), including the H8-mediated VFT1-VFT1 inter-protomer interface, the VFT1-VFT2 intra-protomer interfaces, the VFT2-Ct domains intra-protomer interfaces or the VFT1-Ct domains inter-protomer interfaces, does not markedly affect Bvg kinase activity (Figs. 5 and S5).

Altogether, thus, we have identified interactions in the inter-protomer interfaces between VFT1 and the VFT2 hinge and between VFT2_L2_ and the Ct domain that are necessary to maintain BvgS in its kinase-on state. In particular, the substitutions A_472_A+Y_473_A and W_535_A support the idea that the periplasmic domain exerts a strain on the transmembrane domains, causing the cytoplasmic moiety to adopt a specific conformation corresponding to the kinase-on state. The VFT1s contribute to the strain via the close contacts of their two lobes with the hinges of the tight VFT2 domains. Loosening the periplasmic portion or its connections with the transmembrane helices releases the strain, and therefore the cytoplasmic moiety switches to a distinct, kinase-off state.

### Modulation by nicotinate requires multiple intra-dimer interactions

Negative modulators turn BvgS to the kinase-off state at millimolar concentrations in laboratory conditions, and they possibly mimic *in vivo* ligands that might decrease or turn off virulence genes expression at specific stages of the infection. The sites of interaction of these negative modulators are mostly unknown. We have shown that nicotinate binds to isolated VFT2, even though additional sites cannot be ruled out in the dimer [29], and therefore we used nicotinate to determine how the periplasmic moiety contributes to the response of BvgS to negative modulation. The ability of the BvgS variants described above to respond to nicotinate was thus assessed.

The BvgS_T355C+D442C_ variant with a S-S bond across the VFT2 cavity variant is unresponsive to nicotinate but reverts to the wild type (wt) modulation phenotype when the growth medium is supplemented with TCEP (Figs. 5 and S5). This confirms the *in vivo* formation of the S-S bond and also shows that it, rather than the Cys substitutions, hampers the response to nicotinate. The S-S bond might prevent nicotinate from binding or hamper a conformational changes involved in the response to the negative modulator.

A number of other substitutions similarly abrogate the effect of nicotinate (Figs. 5 and S5). Interestingly, both inter-protomer and intra-protomer interactions are required for BvgS response to negative modulation. These interactions map to the VFT1_L1_-VFT1_L1_, VFT1_L2_-VFT2_L1_, and VFT1_L2_-VFT2_L2_ inter-protomer interfaces and to the VFT1_L2_-VFT2_L1_ and VFT2_L2_-Ct domain intra-protomer interfaces (Figs. 5 and S3). Altogether, a large set of both inter- and intra-protomer interactions is required for the response of BvgS to nicotinate. The fact that the response to negative modulation strongly depends on the integrity of the periplasmic moiety indicates that the transition from the kinase-on state to the kinase-off state implies a concerted conformational change.

### Function of BvgS heterodimers

**Fig 6:**
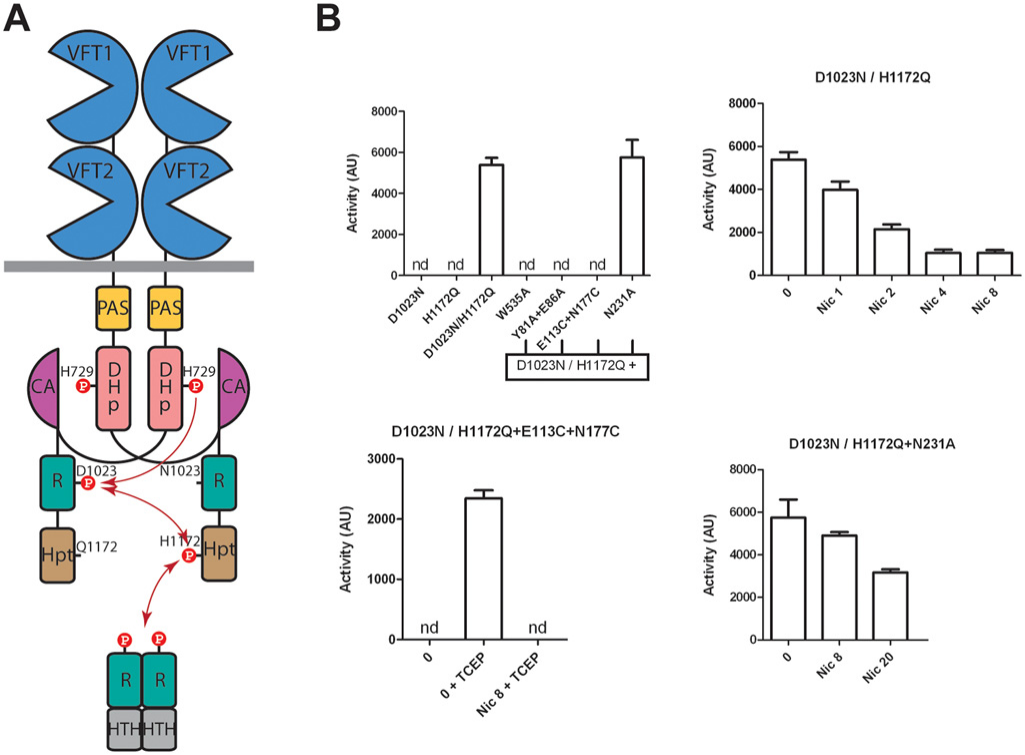
BvgS heterodimers. A. Schematic representation of the BvgS heterodimers. The dimerisation/Histidine phosphotransfer domain (DHp) and the catalytic ATP-binding domain (CA) of the kinase are represented separately to show the phosphorylation cascade (arrows). B. Kinase activity levels as determined using the *ptx-lacZ* reporter for *B*. *pertussis* harboring the indicated BvgS variants and grown in standard or modulation conditions. The first panel shows the activities of the various strains. The first two express inactive homodimers, and the last four express heterodimers in which one protomer harbors a wt periplasmic portion combined with the D_1023_N substitution and the other protomer harbors the indicated periplasmic substitution(s) combined with the H_1172_Q substitution. The last three panels show the β-gal activities of the strains expressing the indicated heterodimers, with the standard errors of the mean calculated from three distinct experiments. Nicotinate (nic) and TCEP were added at the indicated concentrations (in mM). nd, no activity detected.

The importance of the structural integrity of the periplasmic domain for the kinase-on state and for the transition to the kinase-off state was further probed by generating *in vivo* BvgS heterodimers that harbor one wt periplasmic domain and another one with a substitution. A merodiploid containing two inactive but complementary *bvgS* copies, one with a substitution of the phosphorylable Asp of the receiver domain (D_1023_N) and the other with a substitution of the phosphorylable His of the Hpt domain (H_1172_Q), will form inactive homodimers and active heterodimers (Fig. 6) [36,37]. Indeed, only heterodimers will be able to restore the phsophorylation cascade of BvgS. We set up this merodiploid expression system in *B*. *pertussis*. As shown in Fig. 6, the homodimers formed by BvgS_D1023N_ or by BvgS_H1172Q_ are inactive using the *ptx* reporter, but the heterodimer BvgS_D1023N/H1172Q_ is functional, displaying kinase activity in the default state and responding to nicotinate like wt BvgS.

We disrupted critical contacts in one side of the dimer by combining the W_535_A variant with the wt periplasmic protomer. The kinase activity of BvgS was measured using the *ptx-lacZ* system as above. The resulting BvgS homodimer is not functional, similar to the homo-dimeric BvgS_W535A_ variant (Fig. 6). Another variant that harbors the Y_81_A+E_86_A substitutions in the inter-protomer VFT1-VFT2 interface was similarly combined with the wt periplasmic moiety. The heterodimer is also not functional, a phenotype similar to that of the BvgS_Y81A+E86A_ homodimer (Fig. 6). Both results support the model that the periplasmic architecture and more specifically the crucial inter-protomer interfaces identified above impose a kinase-on conformation onto the cytoplasmic moiety via the H19 helices. Releasing the strain in one half of the dimer is sufficient to lose the kinase-on conformation.

We also combined the wt periplasmic moiety with that harboring a S-S bond across the VFT1 cavity. The resulting BvgS heterodimer has no kinase activity (Fig. 6). Thus, both protomers must have the proper conformation and dynamics for BvgS function.

We finally used the heterodimer strategy to test the effect of a substitution that makes BvgS unresponsive to nicotinate. We thus combined a protomer harboring a wt periplasmic domain with that harboring the N_231_A substitution. Asn_231_ from VFT1_L2_ makes interactions with the Ct loop of the other protomer (S3 Fig), and the BvgS_N231A_ homodimer does not respond to nicotinate (Figs. 5 and S5). The recombinant strain expressing the heterodimer has β-galactosidase activity and interestingly, its sensitivity to nicotinate is partially restored. Thus, the heterodimer responds to 20 mM nicotinate, although it is not fully modulated (Fig. 6). This intermediary phenotype indicates that the transition to the kinase-off state requires higher modulator concentrations when the integrity of the periplasmic domain is slightly compromised.

## Discussion

Although the BvgAS system was identified more than 25 years ago [38], the mode of regulation of *Bordetella* virulence has remained a puzzle. With its kinase-on state by default and its extracytoplasmic domain different from those of classical ‘PDC’ (for PhoB/ DcuS/CitA) TCS sensor-kinases, BvgS was initially considered an oddity. However, the realization that many bacterial sensor-kinases harbor similar sensor domains and the first clues about its structure and mode of action have made BvgS a model for the family [23,24,27]. Importantly, some of the BvgS homologs are found in major pathogens, including other *Bordetella* species as well as *P*. *aeruginosa*, *E*. *coli*, *V*. *cholerae*, *Y*. *enterocolitica* and *B*. *burgdorferi*, in which they control programs such as biofilm formation, efflux pump expression, type III secretion, or nutritional adaptation [28–32]. The BvgS structure establishes the foundations to decipher the molecular mode of action of this poorly characterized family of VFT-containing sensor-kinases, and it may pave the way to develop new, highly specific, anti-infective therapeutic strategies [39].

Our functional analyses based on the BvgS structure support the following model. Specific inter-protomer interactions are necessary to maintain the kinase-on state. The tight architecture of the periplasmic moiety together with the differential dynamics of the VFTs imposes a strain onto the transmembrane H19 helices. In response, the cytoplasmic moiety, beginning with the PAS domain, adopts specific conformation and dynamics that support the kinase and phosphotransfer activities of BvgS. The bacteria are thus in the virulent, Bvg^+^ phase, and they can establish an infection. Switching BvgS to the kinase-off state involves a conformational change of the periplasmic moiety, which modifies the conformation, and possibly the dynamics, of the downstream cytoplasmic PAS and kinase domains. The roles of the avirulent or intermediate phases of *B*. *pertussis* are unclear, and *in vivo* stimuli that may trigger the shift to phosphatase or lower kinase states of activity remain to be identified. However, this work shows that the shift to the kinase-off state can easily be hampered by point mutations at various periplasmic sites. That the ability to reversibly perform the shift that regulates BvgS activity has been conserved through evolution supports the importance of the avirulent or intermediate phases in the lifestyle of *B*. *pertussis*. It also strongly argues that the VFT domains perceive negative *in vivo* signals, which explains the good conservation of their cavities in *Bordetella* [35].

As shown in this work, one can artificially turn BvgS to the kinase-off state by disrupting specific inter-protomer interactions between the VFT2 domains and the H19 helices. The release of constraints on these helices causes the cytoplasmic portion to adopt an alternative conformation in which BvgS functions as a phosphatase. We have shown that other events putatively relevant to BvgS function, i.e. the closing of VFT1 domains, which might mimic the binding of a ligand, or the binding of nicotinate to VFT2 [27], also turn BvgS to the kinase-off state. Both most likely cause conformational—and/or dynamic- changes to the periplasmic domain, with repercussions below the membrane. A number of BvgS variants with looser connections between the VFT domains are blocked in the kinase-on state and cannot respond to nicotinate, which shows that the shift to the kinase-off state implies a concerted conformational change. Modulation therefore facilitates the transition by shifting the equilibrium from the kinase-on to the kinase-off conformations. It is likely that these two stable states will also differ in their dynamics, and we have indeed obtained preliminary indications that VFT1 dynamics is modified in the modulated state. Similarly, VFT1 dynamics probably contributes to the transition, in line with the emerging paradigm that the dynamics of signaling proteins relates to their function [40].

In the default situation—i.e., at 37°C and without modulators-, the equilibrium is strongly shifted towards the kinase-on state of BvgS, which is therefore fully populated, while conversely the equilibrium is strongly shifted towards the kinase-off state in the presence of high modulator concentrations. This two-state model is compatible with intermediate levels of activity of the BvgAS system, such as those obtained at intermediate modulator concentrations [15], in which kinase-on and-off BvgS proteins may co-exist in equilibrium. It is also most likely the case for the BvgS_wt/N231A_ heterodimer, in which the lack of a critical interaction on one side of the dimer hampers the transition, and therefore only a proportion of the BvgS molecules shift to the kinase-off state at high modulator concentrations. The transition between the two conformations will likely imply relative rotation, translation or shearing movements of the helices that join the periplasmic and cytoplasmic domains, similar to what has been proposed in other signaling proteins [41–44].

With its clamshell motions, VFT1 behaves like a typical VFT domain. As stated above, the conservation of the VFT1 cavity residues in *Bordetella* [35] suggests that it binds specific ligand(s) *in vivo*, and if so our results show that ligand binding to VFT1 will likely cause BvgS to shift to the kinase-off state. In contrast, the VFT2s remain closed in the kinase-on state with no *bona fide* ligand in their cavity. Whether nicotinate binding to VFT2 opens the cavity or causes another type of deformation remains unknown, but the thermal stabilization of VFT2 upon nicotinate binding argues against the former possibility [27]. The crystal structure of the single VFT domain of a BvgS homolog, the HK29 histidine-kinase of *Geobacter sulfurreducens* interestingly shows that this VFT is also closed unliganded [45]. Its hinge is composed of two β strands, like that of VFT2 in BvgS, leading those authors to propose that it might not be able to open. Sequence analyses of BvgS homologs indicate that the regions forming the hinge of the membrane-proximal VFT domain contain fewer Gly and more Pro residues than those of classical VFT domains. Therefore, we speculate that in the BvgS family the membrane-proximal VFT domain should be closed and tight for the regulation of sensor-kinase activity. BvgS also responds to various organic and inorganic ions [46]. The binding of these modulating molecules might not necessarily involve the cavity but possibly also interfaces, as in some other VFT-containing receptors [47,48].

The periplasmic moiety of BvgS adopts a highly compact dimeric structure. The helical and strongly intertwined architecture of BvgS may explain how some of its homologs could be functional with three, four or even five predicted VFT domains in tandem [24]. The multiple VFT domains of these sensor-kinases potentially enable the perception of several chemical signals that must be integrated to determine the appropriate response. A compact structure like that of BvgS appears to be better suited for inter-domain communication than more linear arrangements such as those found in the VFT-based iGlu receptors of higher eukaryotes, which might dissipate information coming from the most distal VFT domains [49,50] (S6 Fig). This study of BvgS will undoubtedly serve as a basis to elucidate the function of the other family members. Not all BvgS homologs are in a kinase-on state by default [51], but our mechanistic model can perfectly accommodate sensor-kinases that are regulated in the opposite manner.

## Materials and Methods

### Crystallization of BvgS, data collection and processing

The *bvgS* sequence was amplified by PCR and introduced into pGEV2 [52]. The resulting plasmid encodes the periplasmic portion of BvgS (A_29_-L_544_) with N-terminal GB1 and C-terminal His tags. The recombinant protein was purified on a Ni^2+^-Sepharose affinity column (GE Life Sciences) and eluted in 10 mM Tris—HCl (pH 8.8), 500 mM NaCl, 200 mM at 4°C. BvgS was concentrated by ultrafiltration to 20 mg/mL. The initial crystallization screening was carried out using the sitting-drop, vapor-diffusion technique in 96-wells microplates with a Cybi-Workstation (Cybio) and commercial crystallization kits (Nextal-Qiagen and JBSscreen). Extremely fragile crystals were obtained at 19°C by manual refinement in 100 mM sodium acetate (pH 4.6), 1.6 M NaCl, in 5 to 7 days. All manual crystallization attempts were carried out using the hanging-drop, vapor-diffusion technique in 24-well plates. The crystals were soaked in a stepwise fashion to a final concentration of 20% glycerol in the crystallization buffer.

A preliminary diffraction screening was performed on 80 crystals. On the best crystal, diffracting at 3.10 Å, a single diffraction dataset (160 images with an oscillating range of 1°) was collected at an X-ray wavelength of 1.5418 Å and a temperature of 100 K using an in-house Mardtb goniostat and a Mar345 image plate detector. Diffraction images were indexed and scaled using the XDS program package [53]. The crystal belongs to the space group P2_1_2_1_2_1_, with cell parameters a = 72 Å, b = 286 Å and c = 128 Å. According to the calculated Matthews coefficient of 2.52 Å^3^ Da^-1^, a solvent content of 51.3% was estimated.

### Structure determination and refinement

The crystal structure containing four monomers in the asymmetric unit was determined by molecular replacement using MOLREP [54] and the crystallographic structure of the isolated VFT2 domain (PDB code: 3MPK) as a search model. Eight copies of the model were located, four occupying the actual positions of VFT2 domains and the other four those of VFT1 domains, whose sequence identity to VFT2 is 24%. The former four copies were positioned using the conventional Patterson search. The latter four copies were found using an iterative procedure alternating refinement of a partial structure with REFMAC [55] and molecular-replacement search in the electron density maps [56]. Subsequent model rebuilding and refinement of the 3.10 Å structure were conducted iteratively using Coot [57] and phenix.refine [58], with the use of local non-crystallographic symmetry restraints. Torsion angles of the structure were optimized by using the Godzilla web server (http://godzilla.uchicago.edu/) [59]. The structure was refined to final R_work_ of 18.1% and R_free_ of 24.4%. The two BvgS homo-dimers (AB and CD) found in the asymmetric unit can be superimposed with a Cα rmsd of 1.234 Å. A Ramachandran analysis performed with the program Phenix indicated that 94.4% of residues are in preferred conformations and 1.4% in disallowed conformations. The GB1 domains are not seen in the electron density. Analysis of crystal packing revealed an empty space close to the N-terminal segment of each polypeptide chain, indicating that they might be unseen because of crystallographic disorder.

### Structure analyses

The 1026-residue AB dimer was used for all analyses. The opening angles for VFT1 and VFT2 were measured using three residues structurally equivalent between the two VFTs, one on the lip of each lobe and one in the hinge. They are Tyr_70_, Gly_244_ and Ser_199_ for VFT1 and Leu_314_, Glu_490_ and Pro_444_ for VFT2. The inter-domain interfaces were defined using the PISA server (http://www.ebi.ac.uk/pdbe/prot_int/pistart.html) [60], and http://capture.caltech.edu/ was used to identify cation-π interactions. A model for the closed VFT1 domain was made with Modeller [61] based on its closest homologous structure (PDB code: 1WDN), and residues to be replaced by cysteines were chosen by using http://cptweb.cpt.wayne.edu/DbD/ [62].

### Molecular modeling

The methods used for the normal mode analysis and the molecular dynamics simulations with their associated references are described in S1 Protocol. For the analyses of the MD simulations, the opening angles of the VFT domains were calculated based on the geometric centers of the C-α atoms of each lobe and the hinge using a slightly extended definition of the hinge region, encompassing residues 146–151 and 241–246 for that of VFT1, and 390–395 and 486–491 for that of VFT2, to make them less susceptible to noise.

### Measurement of BvgS activity

Point mutations were introduced into the chromosome of *B*. *pertussis* BPSM by allelic exchange [35]. The BvgS sequence corresponds to that of TohamaI except for a Glu residue at position 705, as in most *B*. *pertussis* strains [35]. A *ptx-lacZ* transcriptional fusion was generated in each recombinant strain [63]. The strains were grown in modified Stainer-Scholte medium [64] non-supplemented or containing 1 to 8 mM of nicotinate. Tris(2-carboxyethyl)phosphine (TCEP, SIGMA) was added to 3–10 mM where indicated. TCEP did not affect the activity or the response to nicotinate of wild type BvgS. The bacteria were grown to mid-exponential phase, harvested by centrifugation, resuspended to an OD_600_ of 5 and broken by using a Hybaid Ribolyser apparatus for 30 s at speed 6 in tubes containing 0.1 mm silica spheres. β-galactosidase activities were measured and calculated as described [63]. Each experiment was performed with 3 different clones at different times. The bars represent the standard errors of the mean.

### Detection of inactive BvgS variants

The inactive proteins were detected by immunoblotting of *B*. *pertussis* membrane extracts using anti-BvgS polyclonal antibodies [23] to verify that the substitution(s) generated no major structural defect that might cause BvgS to misfold and to be degraded intracellularly. BPSM_*ΔbvgA*_ and BPMS_*ΔbvgS*_ were described previously [23,35].

### Construction of heterodimers

To construct a *bvgAS* locus deletion strain from BPSM, a *Tohama* I streptomycin- resistant derivative, sequences on either side of the locus (i.e., the 5’ end of the *fhaB* gene and the 3’ end of the *bvgR* gene) were amplified by PCR using the pairs of oligonucleotides iEco-up and Xma-lo, and Xho-up and HindIII-lo (S2 Table). All the amplicons were first introduced into pCRII-TOPO (Invitrogen) and sequenced. The amplicons were introduced as EcoRI-XhoI and XhoI-HindIII fragments into pUC19 by performing a triple ligation, yielding pUC19_newΔbvgAS_. The EcoRI-HindIII insert was then introduced as in [35] into pSORTP1, a mobilizable plasmid for allelic replacement, resulting in BPSM_newΔ_.

The *bvgAS* locus was then constructed as a mosaic gene for allelic replacement in BPSM_newΔ_. We replaced the EcoRI-SpeI part of pUC19_mos_ [35] using a triple ligation with the EcoRI-XmaI fragment obtained as above and a XmaI-SpeI fragment generated using the primers XmaI-up and SpeI-lo. In the latter amplicon, a natural EcoRI site was eliminated by site-directed mutagenesis with a synonymous mutation. The XbaI-HindIII part of pUC19_mos_ was replaced by 3 fragments: a XbaI-NcoI PCR fragment generated using the primers XbaI-up and NcoI-lo, a NcoI-XhoI PCR fragment generated using the primers NcoI-up and XhoI-lo, and the XhoI-HindIII fragment described above. The latter fragment contains a natural NcoI site, which was eliminated as above. The final plasmid was called pUC19_mint_. The 5.5-kb EcoRI-HindIII insert of pUC19_mint_ was transferred into pSORTP1 for allelic exchange.

A plasmidic construction of the *bvgAS* locus was also created starting from pUC19_mint_ and replacing the EcoRI-SpeI fragment by that generated using the primers pEcoRI-up and SpeI-lo. The natural EcoRI site of this latter fragment was eliminated as above. Finally, the NcoI-HindIII fragment of pUC19_mint_ was replaced by another fragment generated using the primers NcoI-up and pHindIII-lo, yielding pUC19_mpla_. The 4.7-kb EcoRI-HindIII insert was transferred into pBBR1-MCS4 [65], a low-copy, mobilizable and replicative plasmid.

The residues Asp_1023_ and His_1172_ were replaced by Asn and Gln, respectively, using site-directed mutagenesis (QuikchangeXL, Agilent). The first mutation was inserted in pUC19_mint_ and then in pSORTP1 for allelic replacement in BPSM_newΔ_. The second mutation was inserted in pUC19_mpla_ and then in pBBR1-MCS4, yielding pBBR_mpla_ to be introduced in *Bordetella* as an episome.

Successive conjugations were then performed to generate the merodiploids. The first one introduced pSORTP1 containing the *bvgAS* locus with the D_1023_N substitution into BPSM_newΔ_, yielding an avirulent strain. Then, pFUS-S1 was integrated to generate the *ptx-lacZ* transcriptional fusion [63], and the resulting strain was finally transformed with pBBR_mpla_ containing *bvgAS* with the H_1172_Q substitution. The mutations of the periplasmic domain were introduced via restriction fragment exchange in pUC19_mpla_ and then in pBBR_mpla_.

### Accession numbers

Atomic coordinates and structure factors for the BvgS periplasmic moiety have been deposited in the Protein Data Bank under the accession number 4Q0C.

## Supporting Information

S1 Table Crystallographic parameters. (DOCX)

S2 Table Oligonucleotides used for the construction of the BvgS heterodimers. (DOCX)

S1 Fig Sequence of the BvgS periplasmic domain and definition of its secondary structure elements. The α helices (H) and β strands (S) are numbered and colored orange and green, respectively. The lobes and hinges between the two lobes of each VFT domain and the Ct loop are also indicated.
(DOCX)

S2 Fig Dynamics of BvgS. A. Amplitude profiles for the first four normal modes of motion based on a Gaussian network model. Fluctuations of the A and B protomers are indicated by black and red curves, respectively. Note that a single mode describes fluctuation probabilities for the dimer. Black and blue horizontal lines delineate VFT1 and VFT2, respectively, with thick lines indicating their lobes 2. B. Distributions of the VFT internal angles over three molecular dynamics simulations. The opening angles of the VFT domains were calculated based on the geometric centers of the Cα atoms of the two lobes and the hinge region, and data were collected every 100 ps. Blue and black curves refer to the VFT2 and VFT1 angles, respectively. The vertical red lines indicate the initial values of the opening angles of the four VFT domains (lower than 110°: VFT2s; higher than 120°: VFT1s). The inset shows a running average of the angles (1-ns window) over the simulations called WT0, WT1 and WT2. The horizontal stippled red lines show the initial opening angles of the four VFT domains.
(DOCX)

S3 Fig Substitutions introduced in BvgS. A. Ribbon representation of the engineered VFT1 and VFT2 Cys variants. The mutated residues are circled in green. The open structure of VFT1 is shown, although the selection of the residues for S-S bond formation was performed using a closed model based on the closest homolog (see Methods). B. Position of the substitutions that make BvgS unresponsive to modulation. One protomer is shown in surface representation, while the other is outlined and colored gray. The pink balls represent the modified residues. A zoom delimited by a dashed orange box shows specific residues whose replacement affects the responsiveness of BvgS to nicotinate but not its kinase activity. Residues Ser_271_ to Ser_275_ are in the α helix H8 that forms the VFT1_L1_-VFT1_L1_ interface. Residues Arg_160_, Phe_230_, Arg_234_, Ser_287_ are in the intra-protomer VFT1-VFT2 interface, and Arg_526_ is in the intra-protomer VFT2-Ct interface. Residues Gln_463_ and Asn_231_ are part of the inter-protomer VFT1-VFT2 and VFT1-Ct interfaces, respectively.
(DOCX)

S4 Fig Detection of specific BvgS variants in membrane extracts of *B*. *pertussis* by immunoblotting. ΔS and ΔA represent strains with deletions of *bvgS* and *bvgA*, respectively. In the right panel in A, the BvgS_E113C+E177C_ band was most likely too faint and fuzzy for detection under non-reducing conditions, but the left panel confirms that the protein is produced and membrane-localized as expected. The amounts of BvgS are generally lower in avirulent strains because the *bvgAS* operon is positively auto-regulated. The asterisk in the right panel denotes that the oxidized BvgS_T355C+D442C_ variant migrates slightly faster than the wild type control. Note that *in vivo* S-S bond formation was confirmed by the observation that the recombinant strain producing the BvgS_T355C+D442C_ variant does not respond to nicotinate modulation, unless the S-S bond is reduced (see S5 Fig). The other non-functional BvgS variants are presented in B, showing that they are all produced and localized in the membrane.
(DOCX)

S5 Fig β-galactosidase activities of recombinant *B*. *pertussis* harboring BvgS variants. The histograms show the β-gal activity levels from the Bvg-regulated *ptx-lacZ* fusion in the respective strains grown in different conditions. Nic indicates the addition of nicotinate to the growth medium at the given concentrations (in mM). TCEP was added to 10 mM to the growth medium where indicated. WT corresponds to the TohamaI strain with the K_705_E substitution in BvgS. The bars represent the standard errors of the mean that were calculated from three different experiments.
(DOCX)

S6 Fig BvgS represents a distinct paradigm of VFT-containing signal-transduction proteins. Cartoon representations compare the structures of an AMPA receptor in A (pdb code: 3KG2), an NMDA receptor in B (pdb code: 4PE5) and of the periplasmic moiety of BvgS in C. The three proteins are shown at the same scale, with each protomer represented in one color. The AMPA and NMDA receptors are tetrameric, with two VFT domains per protomer. The transmembrane segments forming the ion channels are at the bottom of the structure. The extracytoplasmic face of the membrane is represented as a dashed line. For AMPA, the linkers between the NTD (N-terminal domain) and the ABD (agonist-binding domain) and between the ABD and the trans-membrane domain can be seen in the pink and yellow monomers, respectively.
(DOCX)

S1 Protocol *In silico* analyses of BvgS-p dynamics and associated references. (DOCX)
