# Supplementary material for: Virulence Regulation with Venus Flytrap Domains: Structure and Function of the Periplasmic Moiety of the Sensor-Kinase BvgS
Source: PLoS Pathog. 2015 Mar 4;11(3):e1004700. doi: 10.1371/journal.ppat.1004700 (PMC4352136; doi:10.1371/journal.ppat.1004700)
Supplement: S1 Table — (DOCX) [file ppat.1004700.s001.docx]

**Table S1. Crystallographic parameters**

|  | Native |
| --- | --- |
| **Data collection** |  |
| Space group | P2_1_2_1_2_1_ |
| Cell dimensions |  |
| *a*, *b*, *c* (Å) | 72.45, 285.83, 128.16 |
| α, β, γ (°) | 90, 90, 90 |
| Resolution (Å) | 19.99 - 3.1 (3.19-3.10) |
| *R*_sym_ or *R*_merge_ | 20.3 (78.2) |
| *I* / σ*I* | 11.80 (2.68) |
| Completeness (%) | 99.1 (94.5) |
| Redundancy | 6.47 (6.37) |
|  |  |
| **Refinement** |  |
| Resolution (Å) | 19.9- 3.1 |
| No. reflections | 48795 |
| *R*_work_ / *R*_free_ | 18.1 / 24.4 |
| No. atoms |  |
| Protein | 15649 |
| Ligand/ion |  |
| Water |  |
| *B*-factors |  |
| Protein | 44 |
| Ligand/ion |  |
| Water |  |
| R.m.s. deviations |  |
| Bond lengths (Å) | 0.011 |
| Bond angles (°) | 1.452 |

A single diffraction data set was collected from a unique crystal.

The numbers in parentheses represent values for the highest resolution

shell.
