# Supplementary material for: Virulence Regulation with Venus Flytrap Domains: Structure and Function of the Periplasmic Moiety of the Sensor-Kinase BvgS
Source: PLoS Pathog. 2015 Mar 4;11(3):e1004700. doi: 10.1371/journal.ppat.1004700 (PMC4352136; doi:10.1371/journal.ppat.1004700)
Supplement: S2 Table — (DOCX) [file ppat.1004700.s002.docx]

**Table S2. Oligonucleotides for the construction of the BvgS heterodimers**

iEco-up 5’-TAGAATTCGGGTCGGCGATATTGACAACG-3’

Xma-lo 5’-ATCTCGAGCCCGGGGAGCGGGTTGTCGGCGCA-3’

Xho-up 5’-ATCTCGAGCAACGGCCGCATCAAGA-3’

HindIII-lo 5’-ATAAGCTTACTGCAGTCGCCCTATG-3’

XmaI-up 5’-ATCCCGGGCCGCCGCGGGTGC-3’

SpeI-lo 5’-ATACTAGTGACGTGGCGTTCAGCTTCTG-3’

XbaI-up 5’-TTCTAGACATGGGGAAGATCG-3’

NcoI-lo 5’-CGGCCTGCACGGATTTTTCGA-3’

NcoI-up 5’-AACGGTTTCGGTGGACCTGA-3’

XhoI-lo 5’-ATCTCGAGCAACGGCCGCATCAAGA-3’

pEcoRI-up 5’-TAGAATTCAAACCCTGTCCGGCACGTA-3’

pHindIII-lo 5’-ATAAGCTTATGCCGCCCAATTATCCGTA-3’
