# Supplementary material for: Virulence Regulation with Venus Flytrap Domains: Structure and Function of the Periplasmic Moiety of the Sensor-Kinase BvgS
Source: PLoS Pathog. 2015 Mar 4;11(3):e1004700. doi: 10.1371/journal.ppat.1004700 (PMC4352136; doi:10.1371/journal.ppat.1004700)
Supplement: S1 Fig — The α helices (H) and β strands (S) are numbered and colored orange and green, respectively. The lobes and hinges between the two lobes of each VFT domain and the Ct loop are also indicated. (DOCX) [file ppat.1004700.s003.docx]

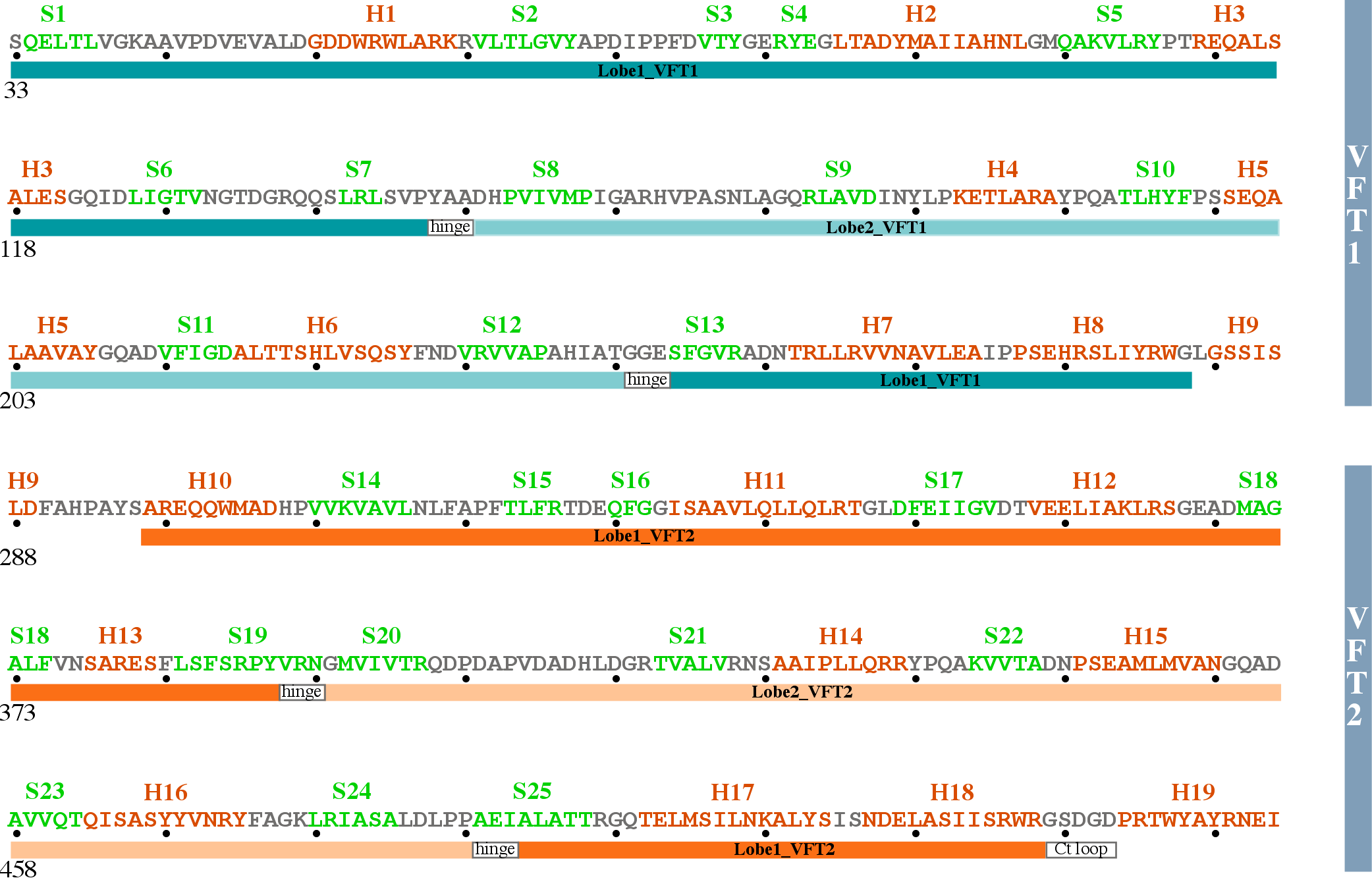


**Figure S1. Sequence of the BvgS periplasmic domain and definition of its secondary structure elements**. The α helices (H) and β strands (S) are numbered and colored orange and green, respectively. The lobes and hinges between lobes are also indicated.
