# Supplementary material for: Virulence Regulation with Venus Flytrap Domains: Structure and Function of the Periplasmic Moiety of the Sensor-Kinase BvgS
Source: PLoS Pathog. 2015 Mar 4;11(3):e1004700. doi: 10.1371/journal.ppat.1004700 (PMC4352136; doi:10.1371/journal.ppat.1004700)
Supplement: S2 Fig — A. Amplitude profiles for the first four normal modes of motion based on a Gaussian network model. Fluctuations of the A and B protomers are indicated by black and red curves, respectively. Note that a single mode describes fluctuation probabilities for the dimer. Black and blue horizontal lines delineate VFT1 and VFT2, respectively, with thick lines indicating their lobes 2. B. Distributions of the VFT internal angles over three molecular dynamics simulations. The opening angles of the VFT domains were calculated based on the geometric centers of the Cα atoms of the two lobes and the hinge region, and data were collected every 100 ps. Blue and black curves refer to the VFT2 and VFT1 angles, respectively. The vertical red lines indicate the initial values of the opening angles of the four VFT domains (lower than 110°: VFT2s; higher than 120°: VFT1s). The inset shows a running average of the angles (1-ns window) over the simulations called WT0, WT1 and WT2. The horizontal stippled red lines show the initial opening angles of the four VFT domains. (DOCX) [file ppat.1004700.s004.docx]

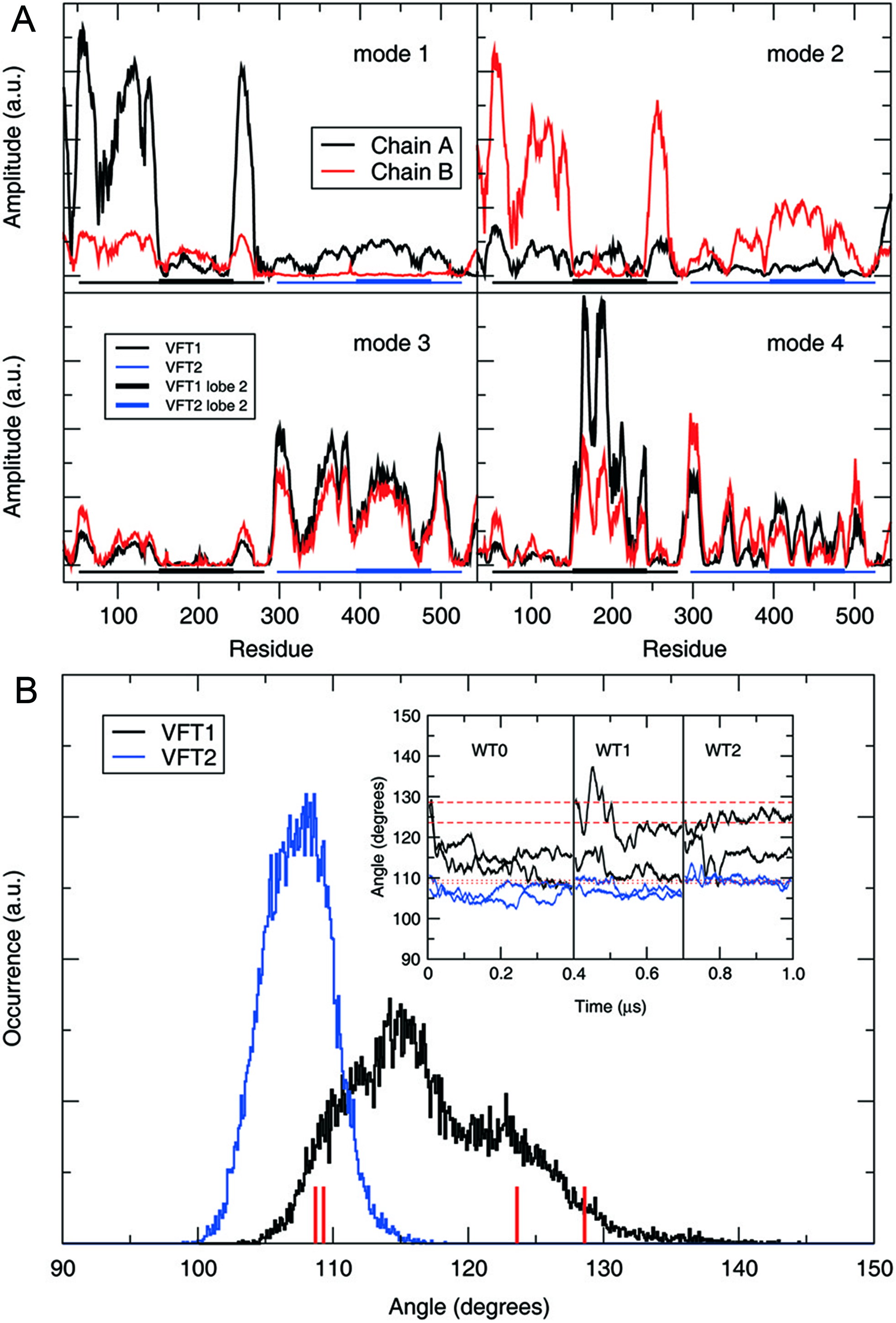


**Figure S2**. **Dynamics of BvgS**. A. GNM amplitude profiles for the first four normal modes. Fluctuations of the A and B protomers are indicated by black and red curves, respectively. Note that a single mode describes fluctuation probabilities for the dimer. Black and blue horizontal lines delineate VFT1 and VFT2, respectively, with thick lines indicating their lobes 2. B. Distributions of the VFT internal angles over the three MD simulations. The opening angles of the VFT domains were calculated based on the geometric centers of the Cα atoms of the two lobes and the hinge region, and data were collected every 100 ps. Blue and black curves refer to the VFT2 and VFT1 angles, respectively. The vertical red lines indicate the initial values of the opening angles of the four VFT domains (lower than 110°: VFT2s; higher than 120°: VFT1s). The inset shows a running average of the angles (1-ns window) over the three MD simulations called WT0, WT1 and WT2. The horizontal stippled red lines show the initial opening angles of the four VFT domains.
