# Supplementary material for: Virulence Regulation with Venus Flytrap Domains: Structure and Function of the Periplasmic Moiety of the Sensor-Kinase BvgS
Source: PLoS Pathog. 2015 Mar 4;11(3):e1004700. doi: 10.1371/journal.ppat.1004700 (PMC4352136; doi:10.1371/journal.ppat.1004700)
Supplement: S3 Fig — A. Ribbon representation of the engineered VFT1 and VFT2 Cys variants. The mutated residues are circled in green. The open structure of VFT1 is shown, although the selection of the residues for S-S bond formation was performed using a closed model based on the closest homolog (see Methods). B. Position of the substitutions that make BvgS unresponsive to modulation. One protomer is shown in surface representation, while the other is outlined and colored gray. The pink balls represent the modified residues. A zoom delimited by a dashed orange box shows specific residues whose replacement affects the responsiveness of BvgS to nicotinate but not its kinase activity. Residues Ser271 to Ser275 are in the α helix H8 that forms the VFT1L1-VFT1L1 interface. Residues Arg160, Phe230, Arg234, Ser287 are in the intra-protomer VFT1-VFT2 interface, and Arg526 is in the intra-protomer VFT2-Ct interface. Residues Gln463 and Asn231 are part of the inter-protomer VFT1-VFT2 and VFT1-Ct interfaces, respectively. (DOCX) [file ppat.1004700.s005.docx]

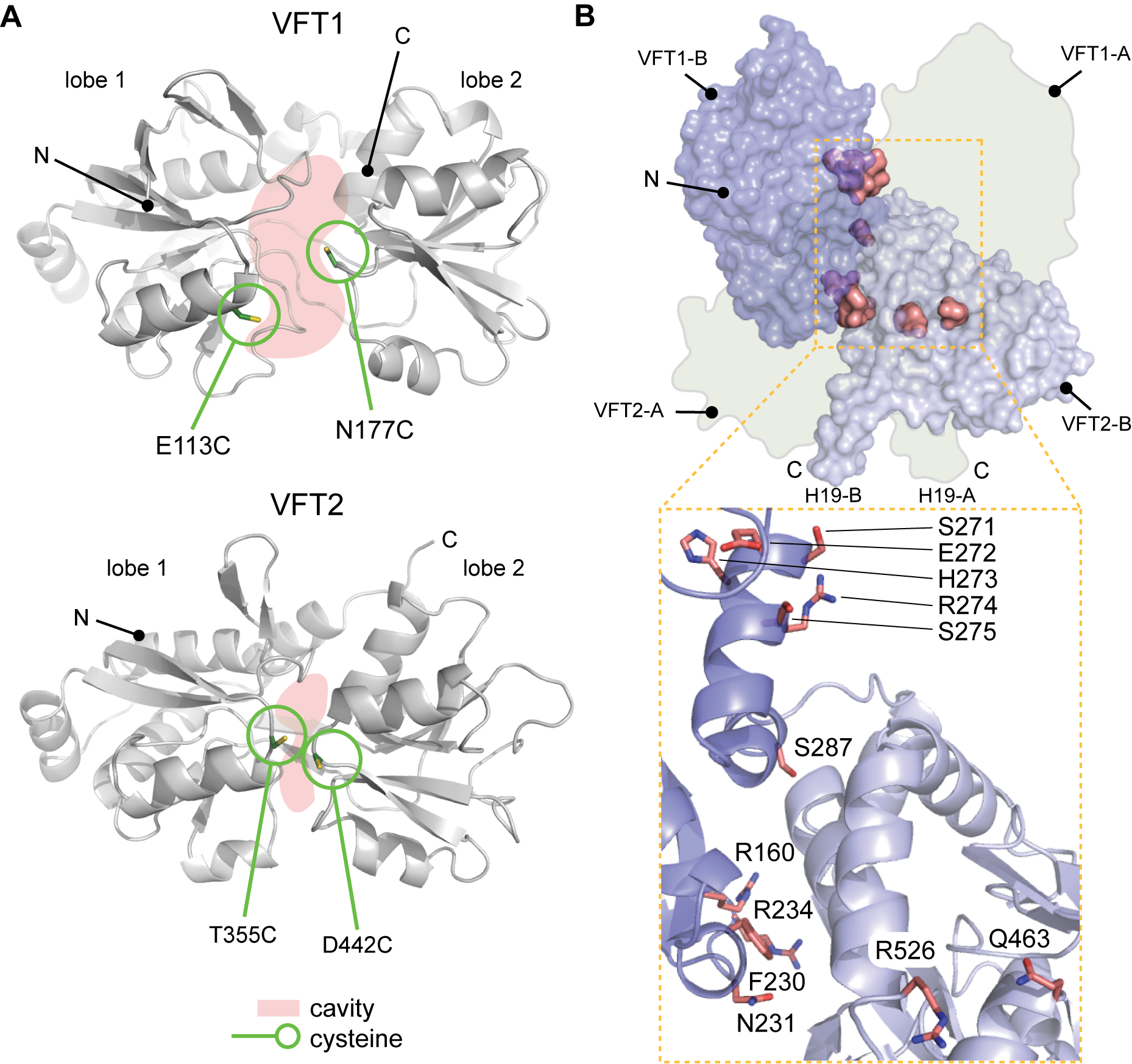


**Figure S3. Substitutions introduced in BvgS.** A. Ribbon representation of the engineered VFT1 and VFT2 Cys variants. The mutated residues are circled in green. The open structure of VFT1 is shown, although the selection of the residues for S-S bond formation was performed using a closed model based on the closest homolog (see Methods). B. Position of the substitutions that make BvgS unresponsive to modulation. One protomer is shown in surface representation, while the other is outlined and colored gray. The pink balls represent the modified residues. A zoom delimited by a dashed orange box shows specific residues whose replacement affects the responsiveness of BvgS to nicotinate but not its kinase activity. Residues Ser_271_ to Ser_275_ are in α helix H8 that forms the VFT1_L1_-VFT1_L1_ interface. Residues Arg_160_, Phe_230_, Arg_234_, Ser_287_ are in the intra-protomer VFT1-VFT2 interface, and Arg_526_ is in the intra-protomer VFT2-Ct interface. Residues Gln_463_ and Asn_231_ are part of the inter-protomer VFT1-VFT2 and VFT1-Ct interfaces, respectively.
