# Supplementary material for: Virulence Regulation with Venus Flytrap Domains: Structure and Function of the Periplasmic Moiety of the Sensor-Kinase BvgS
Source: PLoS Pathog. 2015 Mar 4;11(3):e1004700. doi: 10.1371/journal.ppat.1004700 (PMC4352136; doi:10.1371/journal.ppat.1004700)
Supplement: S4 Fig — ΔS and ΔA represent strains with deletions of bvgS and bvgA, respectively. In the right panel in A, the BvgSE113C+E177C band was most likely too faint and fuzzy for detection under non-reducing conditions, but the left panel confirms that the protein is produced and membrane-localized as expected. The amounts of BvgS are generally lower in avirulent strains because the bvgAS operon is positively auto-regulated. The asterisk in the right panel denotes that the oxidized BvgST355C+D442C variant migrates slightly faster than the wild type control. Note that in vivo S-S bond formation was confirmed by the observation that the recombinant strain producing the BvgST355C+D442C variant does not respond to nicotinate modulation, unless the S-S bond is reduced (see S5 Fig). The other non-functional BvgS variants are presented in B, showing that they are all produced and localized in the membrane. (DOCX) [file ppat.1004700.s006.docx]

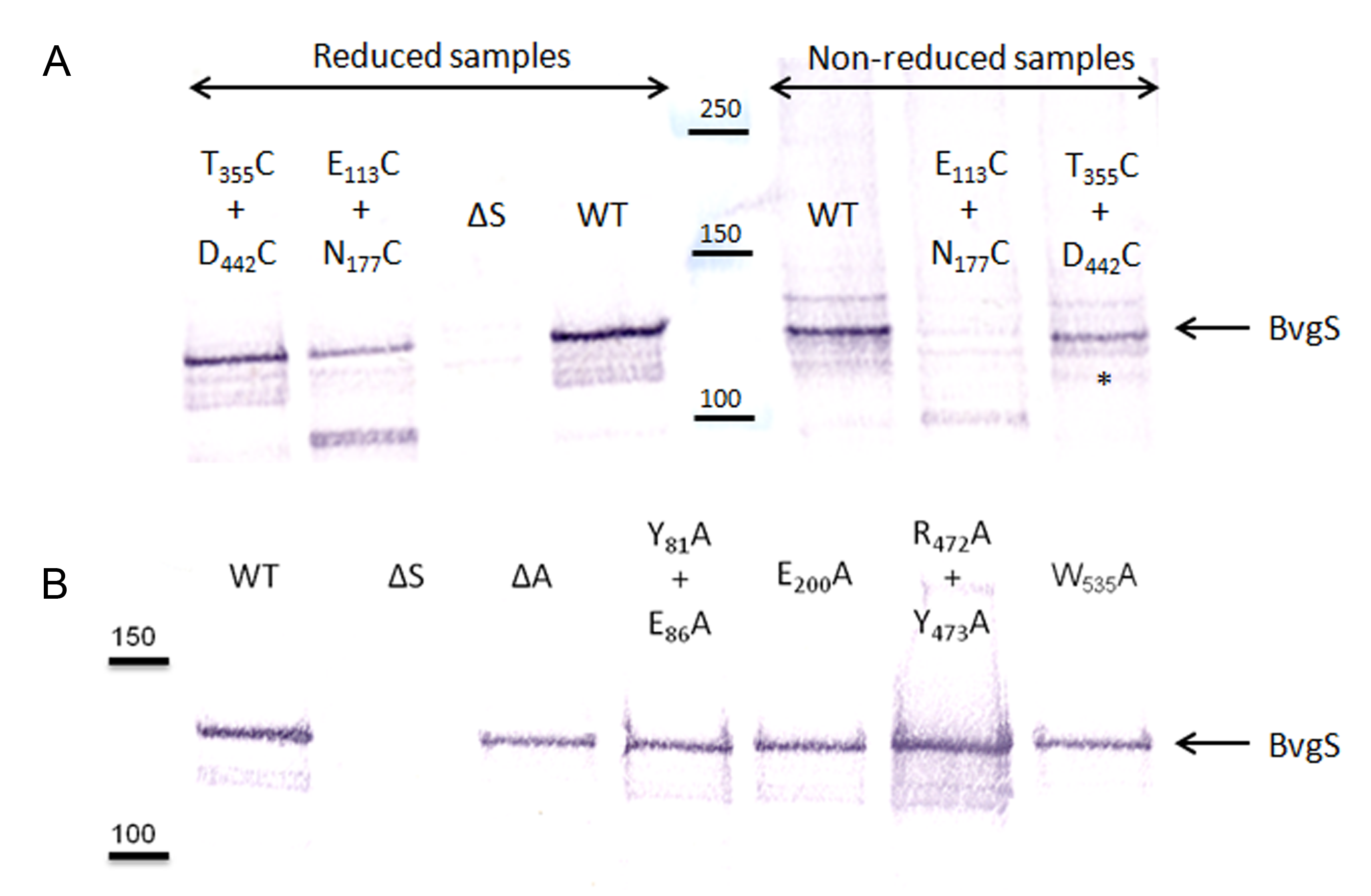


**Figure S4. Detection of specific BvgS variants in membrane extracts of *B. pertussis* by immunoblotting.** ΔS and ΔA represent strains with deletions of *bvgS* and *bvgA*, respectively. In the right panel in A, the BvgS_E113C+E177C_ band was most likely too faint and fuzzy for detection under non-reducing conditions, but the left panel confirms that the protein was produced and membrane-localized as expected. The amounts of BvgS are generally lower in avirulent strains because the *bvgAS* operon is positively auto-regulated. The asterisk in the right panel denotes that the oxidized BvgS_T355C+D442C_ variant migrates slightly faster than the wild type control. Note that *in vivo* S-S bond formation was confirmed by the observation that the recombinant strain producing the BvgS_T355C+D442C_ variant does not respond to nicotinate modulation, unless the S-S bond is reduced (see Fig. S5). The other non-functional BvgS variants are presented in B, showing that they are all produced and localized in the membrane.
