# Supplementary material for: Virulence Regulation with Venus Flytrap Domains: Structure and Function of the Periplasmic Moiety of the Sensor-Kinase BvgS
Source: PLoS Pathog. 2015 Mar 4;11(3):e1004700. doi: 10.1371/journal.ppat.1004700 (PMC4352136; doi:10.1371/journal.ppat.1004700)
Supplement: S5 Fig — The histograms show the β-gal activity levels from the Bvg-regulated ptx-lacZ fusion in the respective strains grown in different conditions. Nic indicates the addition of nicotinate to the growth medium at the given concentrations (in mM). TCEP was added to 10 mM to the growth medium where indicated. WT corresponds to the TohamaI strain with the K705E substitution in BvgS. The bars represent the standard errors of the mean that were calculated from three different experiments. (DOCX) [file ppat.1004700.s007.docx]

**
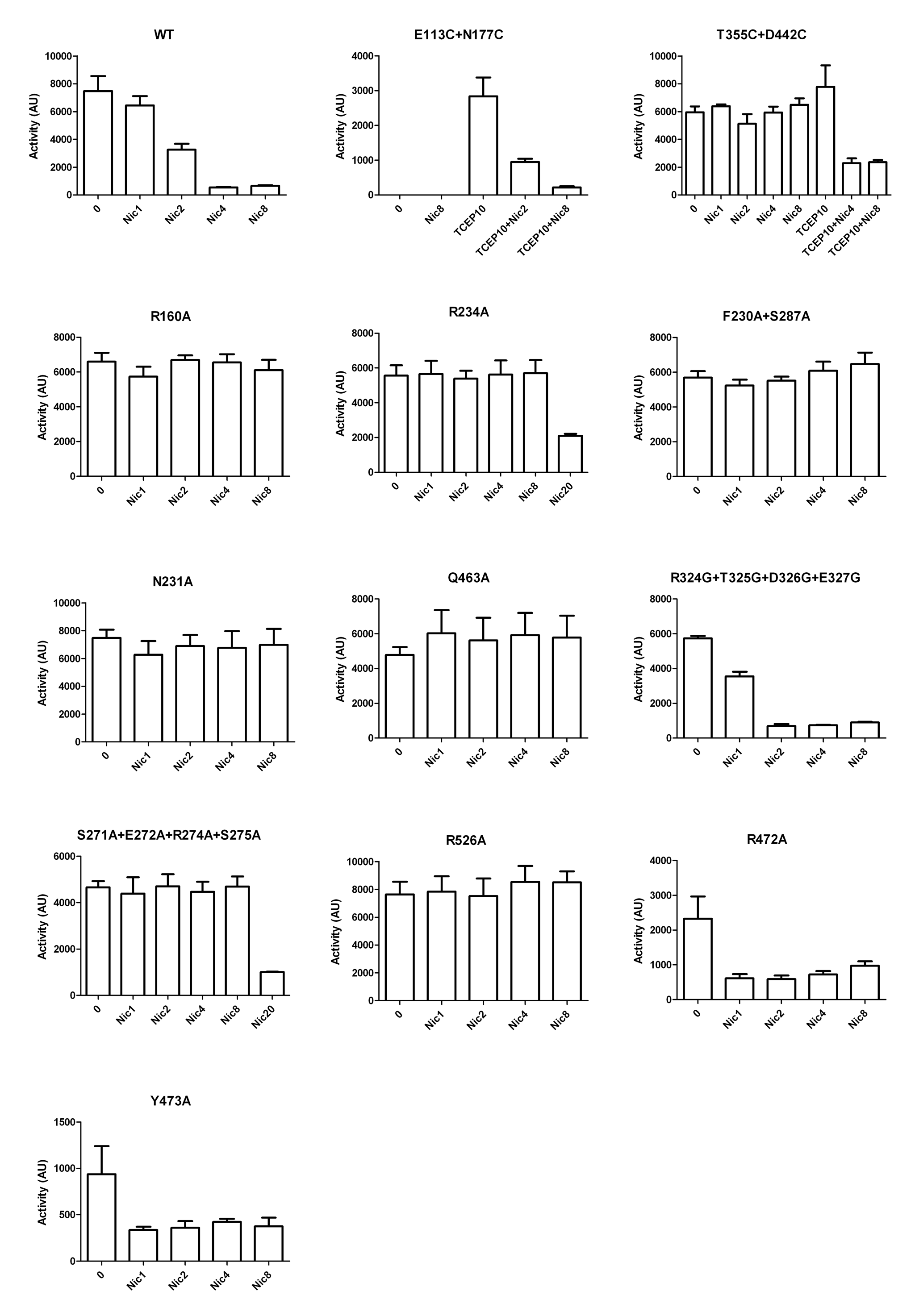
**

**Figure S5**. **β-galactosidase activities of recombinant *B. pertussis* harboring BvgS variants**. The histograms show the β-gal activity levels from the Bvg-regulated *ptx-lacZ* fusion in the respective strains grown in different conditions. Nic indicates the addition of nicotinate to the growth medium at the given concentrations (in mM). TCEP was added to 10 mM to the growth medium where indicated. WT corresponds to the TohamaI strain with the K_705_E substitution in BvgS. The bars represent the standard errors of the mean that were calculated from three different experiments.
