# Supplementary material for: Virulence Regulation with Venus Flytrap Domains: Structure and Function of the Periplasmic Moiety of the Sensor-Kinase BvgS
Source: PLoS Pathog. 2015 Mar 4;11(3):e1004700. doi: 10.1371/journal.ppat.1004700 (PMC4352136; doi:10.1371/journal.ppat.1004700)
Supplement: S6 Fig — Cartoon representations compare the structures of an AMPA receptor in A (pdb code: 3KG2), an NMDA receptor in B (pdb code: 4PE5) and of the periplasmic moiety of BvgS in C. The three proteins are shown at the same scale, with each protomer represented in one color. The AMPA and NMDA receptors are tetrameric, with two VFT domains per protomer. The transmembrane segments forming the ion channels are at the bottom of the structure. The extracytoplasmic face of the membrane is represented as a dashed line. For AMPA, the linkers between the NTD (N-terminal domain) and the ABD (agonist-binding domain) and between the ABD and the trans-membrane domain can be seen in the pink and yellow monomers, respectively. (DOCX) [file ppat.1004700.s008.docx]

**
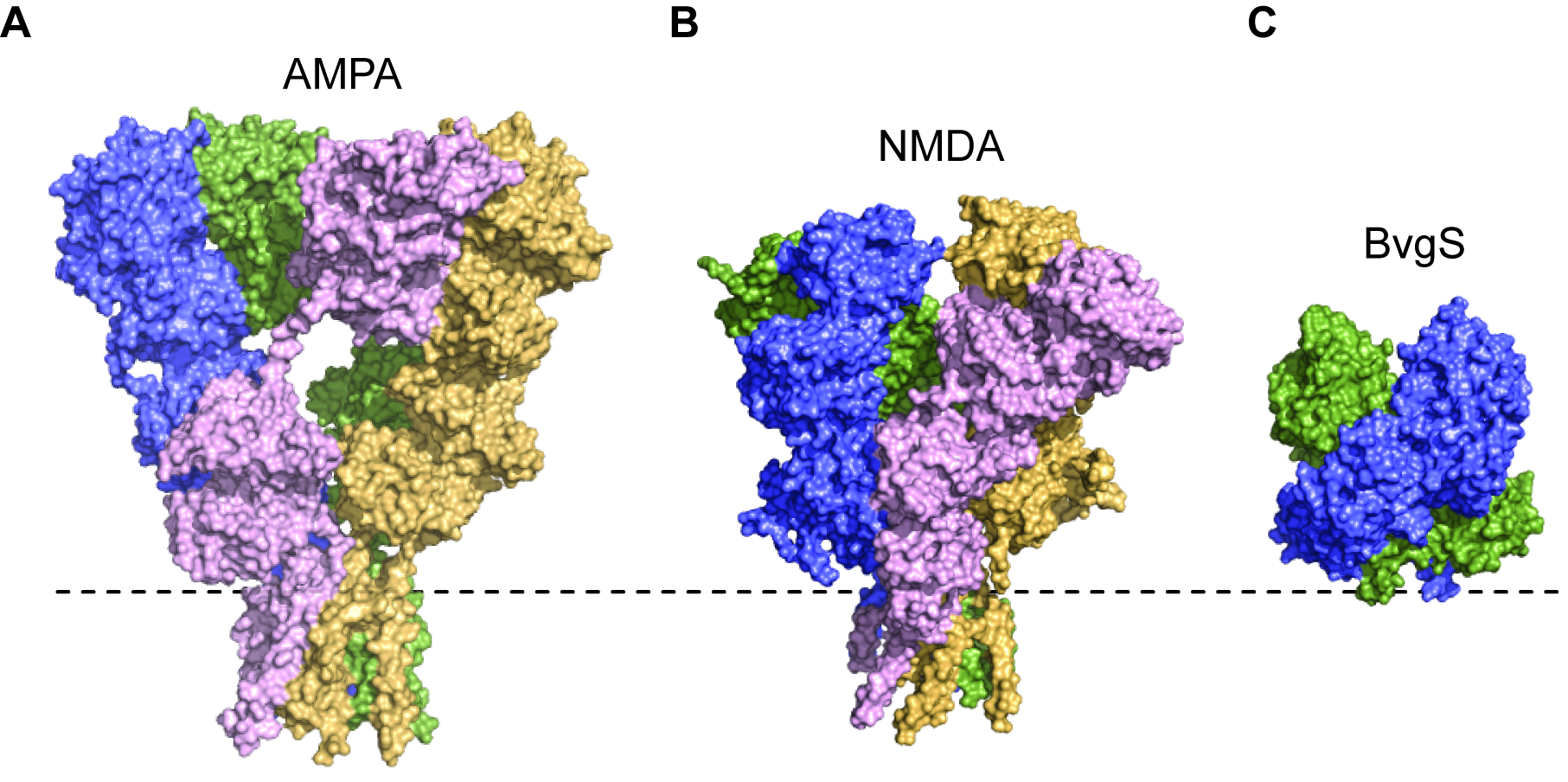
**

**Figure S6. BvgS represents a distinct paradigm of VFT-containing signal-transduction proteins**. Cartoon representations compare the structures of an AMPA receptor in A (pdb code: 3KG2), an NMDA receptor in B (pdb code: 4PE5) and of the periplasmic moiety of BvgS in C. The three proteins all are shown at the same scale, with each protomer represented in one color. The AMPA and NMDA receptors are tetrameric, with two VFT domains per protomer. The transmembrane segments forming the ion channels are at the bottom of the structure. The extra-cytoplasmic face of the membrane is represented as a dashed line. For AMPA, the linkers between the NTD (N-terminal domain) and the ABD (agonist-binding domain) and between the ABD and the trans-membrane domain can be seen in the pink and yellow monomers, respectively.
