# Supplementary material for: Virulence Regulation with Venus Flytrap Domains: Structure and Function of the Periplasmic Moiety of the Sensor-Kinase BvgS
Source: PLoS Pathog. 2015 Mar 4;11(3):e1004700. doi: 10.1371/journal.ppat.1004700 (PMC4352136; doi:10.1371/journal.ppat.1004700)
Supplement: S1 Protocol — (DOCX) [file ppat.1004700.s009.docx]

**Supporting protocol S1.** *In silico* analyses of BvgS-p dynamics and associated references.

Normal mode analyses

Normal mode analyses (NMA) were performed using the standalone version of the HingeProt program [1], using cut-off values of 1.0 nm in the Gaussian Network Model [2].

Molecular dynamics simulations

To prepare the MD simulations, missing side-chain atoms were constructed using the program ‘profix’ of the Jackal package (<http://wiki.c2b2.columbia.edu/honiglab_public/index.php/Software:Jackal>) [3]. The resulting structure file was immersed in a truncated dodecahedron box consisting of SPC water molecules [4], keeping a minimum distance of 1.0 nm from the protein to the edges of the box. Overlapping water molecules were removed, and 22 random water molecules were replaced by Na^+^ atoms to ensure electrostatic neutrality. The resulting system box (volume of 1074.8 nm^3^) contained BvgS, 22 Na^+^ atoms and 30657 water molecules, totaling 102097 particles. The system was equilibrated using 2000 steps of steepest descent energy minimization followed by 100-ps molecular dynamics simulation with weak position restraints on all protein atoms, excluding hydrogens. The calculations were performed with the Gromacs suite of programs [5], version 4.5.4, using the Gromos96 43a2 parameter set [6]. A time step of 2 fs was employed, updating the neighbor list every 5 steps. The system was coupled to a temperature bath at 310 K with a coupling constant of 0.1 ps [7]. Pressure was maintained at 1 bar using isotropic pressure coupling with a coupling constant of 1 ps. Van der Waals interactions were cut off at a distance of 1.0 nm, and electrostatic interactions were calculated with the particle mesh Ewald method [8], using fourth-order splining and a grid spacing of 0.12 nm. Equations of motion for the water molecules were solved analytically with the SETTLE algorithm [9]. All bonds were constrained using the LinCS algorithm [10], and the rotational motion involving CH_3_ groups was slowed down using virtual sites [11]. For the production runs, the time step was increased to 4 fs and the velocity rescaling procedure was used for the temperature coupling [12]. Three production runs were made, one of 400 ns and two of 300 ns. All simulations were run on in-house parallel computing hardware.

Construction of a model of BvgS_E113C/N177C_ with S-S bonded VFT1s

Residues Glu_113_ and Asn_177_ were mutated *in silico* to Cys residues, and the distance between the sulfur atoms was measured. A disulfide bond was created in the molecular topology, and by use of a slow-growth molecular dynamics simulation the bond was slowly decreased to its desired length of 2.04 Å at a rate of 1 Å/ns. A time step of 2 fs was employed. In order to avoid distortion of the protein, initial N-O hydrogen bond donor-acceptor distances *d*_N-O_ in α and β secondary structure elements were measured and subjected to distance-dependent additional restraints by applying a harmonic penalty potential (force constant *k*_dr_) above a chosen threshold distance *d*_r_. For strong hydrogen bonds (*d*_N-O_ < 2.76 Å), a threshold distance *d*_r_ of 2.76 Å with *k*_dr_ = 3 10^6^ kJ/mol/nm was employed, while for weaker hydrogen bonds (2.76 Å < *d*_N-O_ < 3.50 Å) *d*_r_ was increased to 3.50 Å and *k*_dr_ was reduced to 10^6^ kJ/mol/nm. A linear force penalty was applied for distances exceeding 3.4 Å and 5.0 Å for strong and weak hydrogen bonding, respectively. All other simulation parameters were as in the production runs.

**Supporting References**

1. Emekli U, Schneidman-Duhovny D, Wolfson HJ, Nussinov R, Haliloglu T (2008) HingeProt: automated prediction of hinges in protein structures. Proteins 70: 1219-1227.

2. Haliloglu T, Bahar I, Erman B (1997) Gaussian Dynamics of Folded Proteins. Physical Review Lett 79: 3090-3093.

3. Xiang Z, Honig B (2001) Extending the accuracy limits of prediction for side-chain conformations. J Mol Biol 311: 421-430.

4. Berendsen H, Postma J, Van Gunsteren W, Hermans J (1981) Interaction models for water in relation to protein hydration. Intermolecular Forces 11: 331-342.

5. Hess B, Kutzner C, van der Spoel D, E. L (2008) GROMACS 4: Algorithms for Highly Efficient, Load-Balanced, and Scalable Molecular Simulation. J Chem Theory Comput 4: 435–447.

6. Van Gunsteren W, Billeter S, Eising A, Hünenberger P, Krüger P, et al. (1996) Biomolecular simulation: The GROMOS96 manual and user guide. Zürich, Switzerland: Vdf. Hochschulverlag AG an der ETH Zürich.

7. Berendsen H, Postma J, van Gunsteren W, DiNola A, Haak J (1984) Molecular dynamics with coupling to an external bath. J Chem Phys 81: 3684-3690.

8. Essmann U, Perera L, Berkowitz ML, Darden T, Lee H, et al. (1995) A smooth particle mesh Ewald potential. J Chem Phys 103: 8577–8592.

9. Miyamoto S, Kollman P (1992) Settle: An analytical version of the SHAKE and RATTLE algorithm for rigid water models J Comput Chem 13: 952-962.

10. Hess B (2008) P-LINCS: A Parallel Linear Constraint Solver for Molecular Simulation. J Chem Theory Comput 4: 116–122.

11. Feenstra KA, Hess B, Berendsen HJC (1999) Improving efficiency of large time-scale molecular dynamics simulations of hydrogen-rich systems. J Comput Chem 20: 786–798.

12. Bussi G, Donadio D, Parinello M (2007) Canonical sampling through velocity rescaling. J Chem Phys 126: 014101.
